# Skp, Cullin, F-box (SCF)-Met30 and SCF-Cdc4-Mediated Proteolysis of CENP-A Prevents Mislocalization of CENP-A for Chromosomal Stability in Budding Yeast

**DOI:** 10.1371/journal.pgen.1008597

**Published:** 2020-02-07

**Authors:** Wei-Chun Au, Tianyi Zhang, Prashant K. Mishra, Jessica R. Eisenstatt, Robert L. Walker, Josefina Ocampo, Anthony Dawson, Jack Warren, Michael Costanzo, Anastasia Baryshnikova, Karin Flick, David J. Clark, Paul S. Meltzer, Richard E. Baker, Chad Myers, Charles Boone, Peter Kaiser, Munira A. Basrai

**Affiliations:** 1 Genetics Branch, Center for Cancer Research, National Cancer Institute, National Institutes of Health, Bethesda, MD, United States of America; 2 Division of Developmental Biology, Eunice Kennedy Shriver National Institute of Child Health and Human Development, National Institutes of Health, Bethesda, MD, United States of America; 3 Donnelly Centre for Cellular and Biomolecular Research, University of Toronto, Toronto, ON, Canada; 4 Calico Life Sciences LLC, South San Francisco, CA, United States of America; 5 Department of Biological Chemistry, College of Medicine, University of California, Irvine, CA, United States of America; 6 Department of Microbiology and Physiological Systems, University of Massachusetts Medical School, Worcester, MA, United States of America; 7 Department of Computer Science and Engineering, University of Minnesota-Twin Cities, Minneapolis, MN, United States of America; Institute of Functional Epigenetics, GERMANY

## Abstract

Restricting the localization of the histone H3 variant CENP-A (Cse4 in yeast, CID in flies) to centromeres is essential for faithful chromosome segregation. Mislocalization of CENP-A leads to chromosomal instability (CIN) in yeast, fly and human cells. Overexpression and mislocalization of CENP-A has been observed in many cancers and this correlates with increased invasiveness and poor prognosis. Yet genes that regulate CENP-A levels and localization under physiological conditions have not been defined. In this study we used a genome-wide genetic screen to identify essential genes required for Cse4 homeostasis to prevent its mislocalization for chromosomal stability. We show that two Skp, Cullin, F-box (SCF) ubiquitin ligases with the evolutionarily conserved F-box proteins Met30 and Cdc4 interact and cooperatively regulate proteolysis of endogenous Cse4 and prevent its mislocalization for faithful chromosome segregation under physiological conditions. The interaction of Met30 with Cdc4 is independent of the D domain, which is essential for their homodimerization and ubiquitination of other substrates. The requirement for both Cdc4 and Met30 for ubiquitination is specifc for Cse4; and a common substrate for Cdc4 and Met30 has not previously been described. Met30 is necessary for the interaction between Cdc4 and Cse4, and defects in this interaction lead to stabilization and mislocalization of Cse4, which in turn contributes to CIN. We provide the first direct link between Cse4 mislocalization to defects in kinetochore structure and show that SCF-mediated proteolysis of Cse4 is a major mechanism that prevents stable maintenance of Cse4 at non-centromeric regions, thus ensuring faithful chromosome segregation. In summary, we have identified essential pathways that regulate cellular levels of endogenous Cse4 and shown that proteolysis of Cse4 by SCF-Met30/Cdc4 prevents mislocalization and CIN in unperturbed cells.

## Author summary

Genetic material on each chromosome must be faithfully transmitted to the daughter cell during cell division and chromosomal instability (CIN) results in aneuploidy, a hallmark of cancers. The kinetochore (centromeric DNA and associated proteins) regulates faithful chromosome segregation. Restricting the localization of CENP-A (Cse4 in yeast) to kinetochores is essential for chromosomal stability. Mislocalization of CENP-A contributes to CIN in yeast, fly and human cells and is observed in cancers where it correlates with increased invasiveness and poor prognosis. Hence, identification of pathways that regulate CENP-A levels will help us understand the correlation between CENP-A mislocalization and aneuploidy in cancers. We used a genetic screen to identify essential genes for Cse4 homeostasis and identified a major ubiquitin-dependent pathway where both nuclear F-box proteins, Met30 and Cdc4 of the SCF complex, cooperatively regulate proteolysis of Cse4 to prevent its mislocalization and CIN under physiological conditions. Our studies define a role for SCF-mediated proteolysis of Cse4 as a critical mechanism to ensure faithful chromosome segregation. These studies are significant because mutations in human homologs of Met30 (β-TrCP) and Cdc4 (Fbxw7) have been implicated in cancers, and future studies will determine if SCF-mediated proteolysis of CENP-A prevents its mislocalization for chromosomal stability in human cells.

## Introduction

The kinetochore serves as a site for microtubule attachment and facilitates the separation of sister chromatids during mitosis for high fidelity chromosome segregation. Despite the divergence in DNA sequences, kinetochores in most species contain an evolutionarily conserved histone H3 variant (Cse4 in *Saccharomyces cerevisiae*, Cnp1 in *Schizosaccharomyces pombe*, CID in *Drosophila melanogaster*, and CENP-A in humans), which is essential for centromere (*CEN*) identity, kinetochore assembly and faithful chromosome segregation [1, 2]. Overexpression of centromere protein-A (CENP-A) results in mislocalization to non-centromeric chromosomal regions and contributes to chromosomal instability (CIN) in yeast, fly and human cells [3–8]. Overexpression and mislocalization of CENP-A has been observed in many cancers and correlates with increased invasiveness and poor prognosis [9–14]. However, the molecular mechanisms for this correlation are not understood. Hence, identification of pathways that regulate the cellular levels of CENP-A are critical to understand how high levels of CENP-A contribute to its mislocalization and aneuploidy in cancers.

Ubiquitin-proteasome pathways play a critical role in the regulation of cellular levels of Cse4 and its homologs in order to prevent mislocalization to non-centromeric chromatin in budding yeast, fission yeast and flies [15–19]. Ubiquitination of substrates for proteasome-mediated degradation is catalyzed by three classes of enzymes, namely the E1 ubiquitin-activating enzyme, E2 ubiquitin-conjugating enzyme and E3 ubiquitin ligase [20–22]. Studies with budding yeast have identified the non-essential E3 ubiquitin ligase Psh1, Sumo-targeted ubiquitin ligases (STUbLs) Slx5, Slx8 and the Skp-Cullin-F-box (SCF)-Rcy1 in ubiquitin-mediated proteolysis of overexpressed Cse4 [23–28]. Both the N-terminus and the CENP-A targeting domain (CATD) in the C-terminus of Cse4 are required for Psh1-mediated proteolysis of overexpressed Cse4 [19, 24, 25]. Psh1-mediated proteolysis of Cse4 is also regulated by the FACT (Facilitate Chromatin Transcription/transactions) complex and Casein kinase 2 (CK2) [29, 30]. In addition to ubiquitin ligases, chromatin associated complexes, such as the SWI/SNF, HIR and kinetochore protein Spt4, prevent mislocalization of Cse4 to non-centromeric regions [31–34]. Recently, it was shown that the cell cycle regulated expression of Cid and Cnp1 contribute to preventing their mislocalization to non-centromeric regions in flies and fission yeast, respectively [35, 36].

Cse4 is not completely stabilized in a *psh1Δ rcy1Δ slx5Δ ubr1Δ* quadruple mutant [37], which suggests the presence of additional pathways that regulate cellular levels of Cse4. Major defects in Cse4 proteolysis are expected to compromise viability due to severe CIN, but essential genes for this regulation have not been reported thus far. Hence, we performed a genome-wide screen using a Synthetic Genetic Array (SGA) of temperature sensitive (TS) alleles for 560 essential genes to identify mutants that exhibit Synthetic Dosage Lethality (SDL) when Cse4 is overexpressed [38–41]. The screen revealed 160 alleles that displayed significant growth inhibition with overexpressed Cse4. Gene Ontology (GO) analysis of these genes revealed an enrichment of components involved in ubiquitin-proteasome pathways, especially components of the SCF-ubiquitin ligase complexes with the F-box proteins Met30 and Cdc4 [22].

SCF-ubiquitin complexes are among the best characterized subgroup of Cullin-Ring ligases (CRLs) which represent the largest class of E3 enzymes. The SCF ubiquitin E3 ligase complex is comprised of the core components Skp1, Cullin-1 (Cdc53) and the variable substrate-specifying F-box protein subunits. These components assemble into a functional complex with Rbx1, a RING domain-containing protein, which interacts with the E2 conjugating enzyme (Cdc34) that catalyzes the transfer of ubiquitin moieties to the substrate [42]. SCF-mediated ubiquitination of substrates regulates a range of cellular pathways including cell cycle progression, signal transduction and transcription [43]. Yeast encodes 22 different F-box proteins [22]. Notably, Met30 and Cdc4 are the only essential F-box proteins that form active ubiquitin ligases [22, 44]. Met30 coordinates cell division with nutrient or heavy metal stress by ubiquitination and inactivation of its main target, the transcriptional regulator Met4 [45–48]. Ubiquitinated Met4 functions as a receptor for SCF-Met30/Met4 and triggers the ubiquitination and degradation of several Met4 binding factors such as the cell cycle regulator Met32 [49]. Cdc4 has roles in the cell cycle, cell metabolism and epigenetics by regulating ubiquitin-mediated proteolysis of targets such as the cyclin-dependent kinase inhibitor Sic1 [50], the transcription factor Gcn4 [51], and the histone deacetylase Hst3 [44, 52, 53].

In the present study, we identified the two essential SCF ubiquitin ligases defined by the F-box proteins Met30 and Cdc4 as major regulators of Cse4 proteolysis and localization. We show that Met30 and Cdc4 cooperatively regulate Cse4 proteolysis under normal physiological conditions. Together, our results suggest SCF-Met30/Cdc4-mediated proteolysis of Cse4 is one of the major mechanisms that prevents stable maintenance of Cse4 at non-centromeric regions, thus ensuring faithful chromosome segregation.

## Results

### A genome–wide screen reveals an essential role of proteasomal degradation and ubiquitin ligase activity for growth when Cse4 is overexpressed

Major pathways that prevent Cse4 mislocalization to non-centromeric regions are critical to prevent CIN, and we therefore expected such pathways to be essential for viability of haploid yeasts. To sensitize a genetic approach for identification of these pathways, we used strains with temperature sensitive (TS) alleles of essential genes to identify those that display SDL when Cse4 is overexpressed (*GAL-CSE4*). A query strain with *GAL-CSE4* integrated at the endogenous *CSE4* locus was mated to an array of 786 conditional TS mutant strains, representing 560 essential genes, and deletions of 186 non-essential genes for internal calibration of the SGA interaction score. Growth of the haploid meiotic progeny of each mutant with *GAL-CSE4* was scored on galactose medium at the permissive growth temperature of 26°C. The SGA score for growth was determined as previously described [38] and filtered using the intermediate confidence threshold (*p*-value <0.05 and |Score| >0.08) [39, 40] (S1 Table).

**Fig 1:**
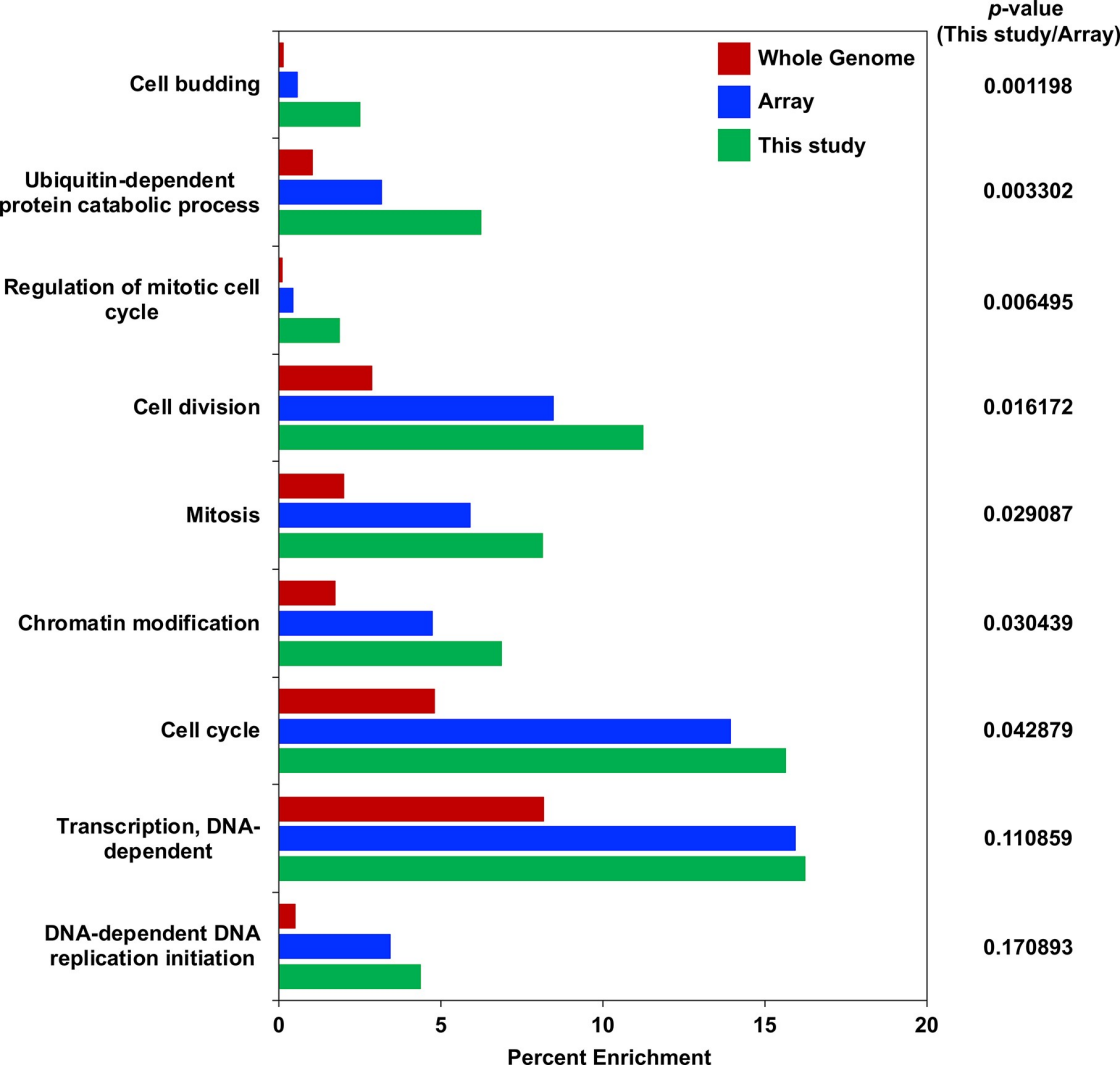
GO analysis of negative genetic interactor genes with *GAL-CSE4* for biological processes. Enrichment of genes in biological processes. The GO analysis for biological processes was performed (*p*-values ranging from 7.72e^-09^ to 1.74e^-04^). Displayed is the percentage of annotated genes in each category over the number of genes in the whole genome (Red bars), genes from the TS array in each category over the number of genes present on the TS array (Blue bars), and genes in each category identified from This study over the total number of negative genetic interactors (Green bars). The order of biological process groups is arranged based on the calculated p value which assesses the probability of having a genetic interaction with *GAL-CSE4* in a given biological process from the genes available on the TS array (most significant on the top).

Three biological replicates of the SGA screen identified 160 alleles representing 140 genes that exhibited significant growth inhibition with *GAL-CSE4* and are referred to as negative genetic interactors. Gene Ontology (GO) analysis for molecular functions and cellular components identified categories related to the proteasome complex, SCF ubiquitin ligase complex, ubiquitin binding, ubiquitin-protein ligase activity, chromatin and nucleotide binding and ATPase activity (*p*-value ranging from 7.72e^-09^ to 9.00e^-03^) (Table 1). We also performed GO analysis of the negative genetic interactors for biological processes (Fig 1). This revealed an enrichment of genes that regulate cell budding, ubiquitin-dependent protein catabolic process, mitotic cell cycle, cell division, chromatin modification, transcription and DNA-dependent replication initiation. Given that the TS array only represents a fraction of the whole genome, we examined the relative enrichment of the negative genetic interactors (This study) to genes in a given category on the TS array (Array) (Fig 1). These results confirm the importance of biological processes such as cell budding, ubiquitin-dependent protein catabolic process and regulation of mitotic cell cycle in the cells overexpressing Cse4. Moreover, the majority of the negative interactor genes (105 of the 113 genes) are evolutionarily conserved with homologs in human, mouse, flies and/or worms (S1 Table). Based on cross-species studies, 57 yeast mutants are complemented by human homologs (S1 Table). Overall, analysis of the SGA screen for SDL with *GAL-CSE4* in essential gene mutants resulted in an enrichment of genes encoding for ubiquitin-dependent catabolic processes, proteasome degradation pathway and ubiquitin ligase activity (Table 1 and Fig 1).

**Table 1:** Gene Ontology (GO) analysis of the negative genetic interactors for molecular functions and cellular component when Cse4 is overexpressed.

GO Term	Gene Name	Fraction	*p*-value
**Molecular Function**			
Ubiquitin binding	*CDC4 CKS1 DOA1 MET30 TAF5 UBC4*	6/37	1.10e^-04^
Structural molecule activity	*NUP57 NUP145 PDS5 RPN6 RPN7 RPN10 SEC13*	7/63	3.40e^-04^
tRNA (adenine-N1-)-methyltransferase activity	*GCD10 GCD14*	2/2	4.46e^-04^
ATP binding	*ARO1 ARP2 ARP3 CAB5 CCA1 CCT6 CDC7 CDC15 CDC48 DED1 MOT1 MPS1 MYO2 ORC1 PRI1 RFC5 RRP3 SEC18 SLM3 SMC1 SMC4 UBA4 UBC4 VAS1 YDJ1 YTA7*	26/622	5.28e^-04^
Nucleotidyltransferase activity	*CCA1 POL1 POL3 PRI1 QRI1 RPO31*	6/49	5.39e^-04^
Binding	*ARO1 CDC27 CDC23 CDC48 CLF1 ETR1 MOT1 PDS5 PRP6 RPN1 RPN6 RPN7 SCC2 SEC18 STU1 UBA4*	16/300	5.68e^-04^
Endopeptidase activity	*PRE2 PRE4 RPN1 SCL1*	4/20	7.20e^-04^
Ubiquitin-protein ligase activity	*APC11 CDC4 CDC23 CDC27 CDC36 CDC53 UBC4*	7/77	1.16e^-03^
**Cellular Component**			
Nucleus	***APQ12 ASA1*** *ASF1* ***BRL1*** *CAB5 CCA1 CDC4 CDC7 CDC14 CDC23 CDC27 CDC36 CDC48 CDC53* ***CIK1*** *CKS1 CLF1 DCP2 DOA1 ELP4 FIP1 GCD10 GCD14 GLC7 GLE1 HRP1 HSF1 IKI3* ***LGE1*** *MET30 MOT1 MRE11* ***NBP2*** *NIP7 NOP2 NOP56 NUP57 NUP145 ORC1 PCF11 PDS5 PHO80 POL1* ***POL3*** *PRE2 PRE4 PRP6 PRP18 QRI1 RFC5 RIM20 RNA15 RPC34 RPN1* ***RPN4*** *RPN7 RPN11 RPO31 RRP3 RSC8* ***RTT109*** *SAP30 SCC2 SCL1 SDS3 SEC13* ***SLD3*** *SMC1 SMC4* ***SMI1*** *SPC110 SPT3 SRM1* ***STB5 STP1*** *STS1 STU1 SWC4 TAF5 TAF12 TFB1* ***TFC8*** *TIF6 TPT1 URM1 VPS71 YKE2 YTA7 ZPR1*	89/1965	1.03e^-14^
Proteasome complex	*PRE2 PRE4 RPN1 RPN5 RPN6 RPN7 RPN10 RPN11 RPN12 SCL1 UBC4*	11/46	1.86e^-09^
Proteasome storage granule	*PRE2 PRE4 RPN1 RPN5 RPN6 RPN11 RPN12 SCL1*	8/26	3.78e^-08^
Proteasome regulatory particle, lid subcomplex	*RPN5 RPN6 RPN7 RPN11 RPN12*	5/10	9.23e^-07^
Nuclear SCF ubiquitin ligase complex	*CDC4 MET30*	2/2	4.45e^-04^

The 140 significant negative genetic interactor genes were analyzed (http://funspec.med.utoronto.ca/, April 2017; *p*-value cutoff = 0.01) for GO term analysis for molecular functions and cellular components. Listed are the GO categories with *p*-values ranging from 1.03e^-14^ to 1.16e^-03^, gene names, and fraction of genes from the input over the total number in a given category. All genes except the ones in bold letters are evolutionarily conserved.

### Mutants of SCF-Met30 and SCF-Cdc4 exhibit SDL with overexpressed Cse4

**Fig 2:**
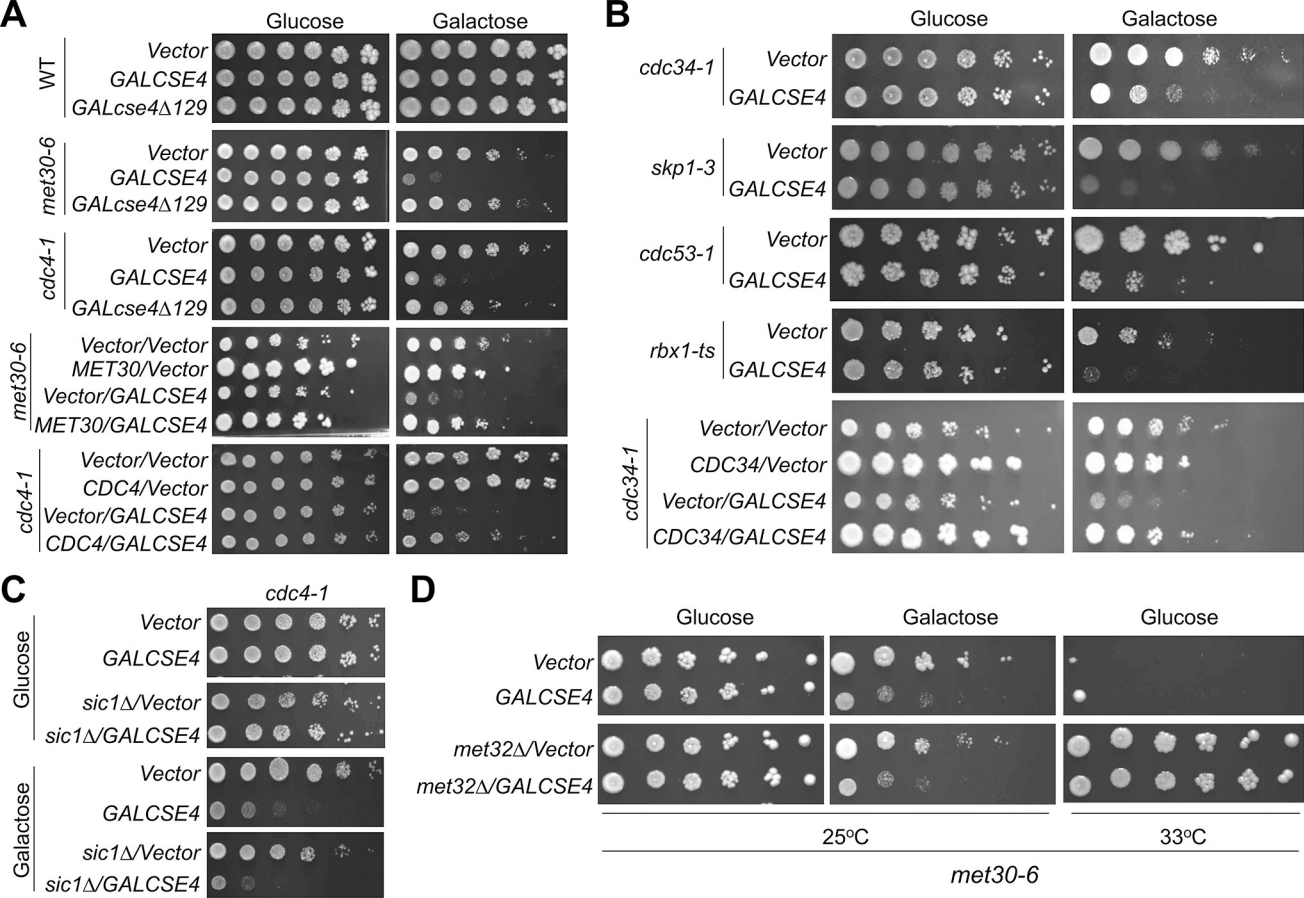
SCF-Cdc4 and SCF-Met30 mutants display SDL with *GAL-CSE4*. (A) *met30* and *cdc4* mutants display SDL with *GAL-CSE4*. WT (BY4741) or the indicated mutant strains transformed with vector (pMB433), *GAL-HA-CSE4* (pMB1597) or *GAL-HA-cse4Δ129* (pMB1459) were grown to logarithmic phase, five-fold serial dilutions were prepared and plated on either glucose or galactose plates at 25°C. Complementation of *GAL-CSE4* induced SDL of *met30-6* and *cdc4-1* was performed with *met30-6* (TSA948) and *cdc4-1* (TSA878) strains with or without *GAL-CSE4* transformed with vector or plasmids expressing *MET30* (pMB1619) or *CDC4* (pMB1617) at 25°C. (B) Mutants of SCF components display SDL with *GAL-CSE4*. WT (BY4741) or the indicated mutant strains transformed with vector (pMB433) or *GAL-HA-CSE4* (pMB1597) were grown to logarithmic phase; five-fold serial dilutions were prepared and plated on either glucose or galactose plates at 25°C except for the *cdc53-1* strain, which was incubated at 33°C. Complementation of *GAL-CSE4* induced SDL of *cdc34-1* was performed with *cdc34-1* with or without *GAL-CSE4* transformed with vector or plasmid expressing *CDC34* (pMB1618) at 25°C. (C) *sic1Δ* does not rescue the SDL of *GAL-CSE4 cdc4-1*. Growth assays were performed using *cdc4-1* (YMB9571) and *cdc4-1 sic1Δ* (YMB9713) with or without *GAL-CSE4* plated on glucose or galactose plates at 25°C. (D) *met32Δ* does not rescue the SDL of *GAL-CSE4 met30-6* strain. Growth assays were performed using *met30-6* (YMB8442) and *met32Δ met30-6* (YMB10681) strains with or without *GAL-CSE4* (pMB1597) plated on glucose or galactose medium at 25°C. The suppression of temperature sensitivity of *met30-6* by *met32Δ* was tested on glucose medium at the non-permissive temperature of 33°C.

The SGA screen identified the SCF ubiquitin ligase complex components Cullin-1/Cdc53 and both nuclear F-box proteins, Met30 and Cdc4 (Table 1 and Fig 1). To confirm the SGA results and further investigate the role of the SCF complex in proteolysis of Cse4, we transformed *cdc53-1*, *met30-6* and *cdc4-1* strains with a *GAL-CSE4* plasmid or empty vector and assayed for growth on plates containing glucose or galactose. The *cdc53-1*, *met30-6* and *cdc4-1* strains exhibit SDL with *GAL-CSE4* on galactose plates at the permissive temperature of 25°C (Fig 2A and 2B). No growth defects in the strains transformed with vector alone on galactose plates were observed (Fig 2A and 2B). Flow cytometry analysis showed that logarithmically grown *met30-6* and *cdc4-1* strains did not exhibit defects in cell cycle progression at 25°C (S1 Fig), excluding that cell cycle position effects are responsible for the genetic interaction. The *GAL-CSE4*-mediated SDL in these mutants was linked to mutations in *MET30* or *CDC4* as the growth defects of *met30-6* and *cdc4-1* with *GAL-CSE4* were partially suppressed by expressing their respective WT genes in these strains (Fig 2A). The lack of complete suppression may be due to the presence of the defective mutant protein that may compete with the wild type protein for binding to Cse4 or Skp1. We also examined the SDL phenotype with an E2 enzyme mutant (*cdc34-1*), as well as alleles for *SKP1* (*skp1-3)* and *RBX1* (*rbx1-ts*) genes that encode the remaining components of the SCF complex [42] which were not included on the TS array. Growth assays showed that *cdc34-1*, *skp1-3* and *rbx1-ts* strains exhibit SDL with *GAL-CSE4* on galactose plates at the permissive temperature (Fig 2B). Expression of *CDC34* suppressed the SDL of *GAL-CSE4 cdc34-1* cells (Fig 2B).

We next examined if the N-terminus of Cse4 is required for the SDL of *GAL-CSE4* in *met30-6* and *cdc4-1* strains. The rationale for this is based on the essential role of the N-terminus for its interactions with kinetochore proteins, Ub-mediated proteolysis and post-translational modifications (PTMs) of Cse4 [19, 26, 27, 54–62]. Furthermore, we recently showed that *hir* mutants, which are defective in proteolysis of overexpressed Cse4, are sensitive to *GAL-CSE4* but not *GAL-cse4Δ129* (Cse4 lacking the N-terminal domain) [33]. Growth assays showed that *GAL-cse4Δ129* did not result in lethality in WT, *met30-6* or *cdc4-1* strains (Fig 2A), suggesting that the N-terminus of Cse4 is required for the SDL of *GAL-CSE4*.

Previous studies have defined roles for SCF-Cdc4 in ubiquitination of cellular substrates, with the cell cycle inhibitor Sic1 being the most critical one [50]. Accordingly, deleting *SIC1* suppresses the G1-S transition defect of a *cdc4-1* strain, but *cdc4-1 sic1Δ* double mutants arrest at later stages in the cell cycle [53]. SCF-Met30 ubiquitinates Met4 and Met32 in a Met4-dependent manner, and deletion of *MET4* or *MET32* suppresses the temperature sensitivity of *met30-6* strains [47, 49]. Therefore, we determined if deletion of *SIC1* or *MET32* would suppress the SDL phenotype of *GAL-CSE4* in *cdc4-1* and *met30-6* strains, respectively. Growth assays showed that the SDL of *GAL-CSE4* in *cdc4-1* cells remained unaffected when combined with *sic1Δ* (Fig 2C). Similarly, the SDL of *GAL-CSE4 met30-6* was not suppressed by *met32Δ*. As expected, the temperature sensitivity of *met30-6* strain was suppressed in the *met32Δ met30-6* strain at 33°C (Fig 2D). These results show that SCF-Met30 and SCF-Cdc4 complexes are essential for growth when Cse4 is overexpressed and that the SDL of *GAL-CSE4* in *cdc4-1* and *met30-6* strains is independent of the key targets of Cdc4 and Met30.

### Met30 and Cdc4 interact with Cse4 and regulate ubiquitin-mediated proteolysis of overexpressed Cse4

**Fig 3:**
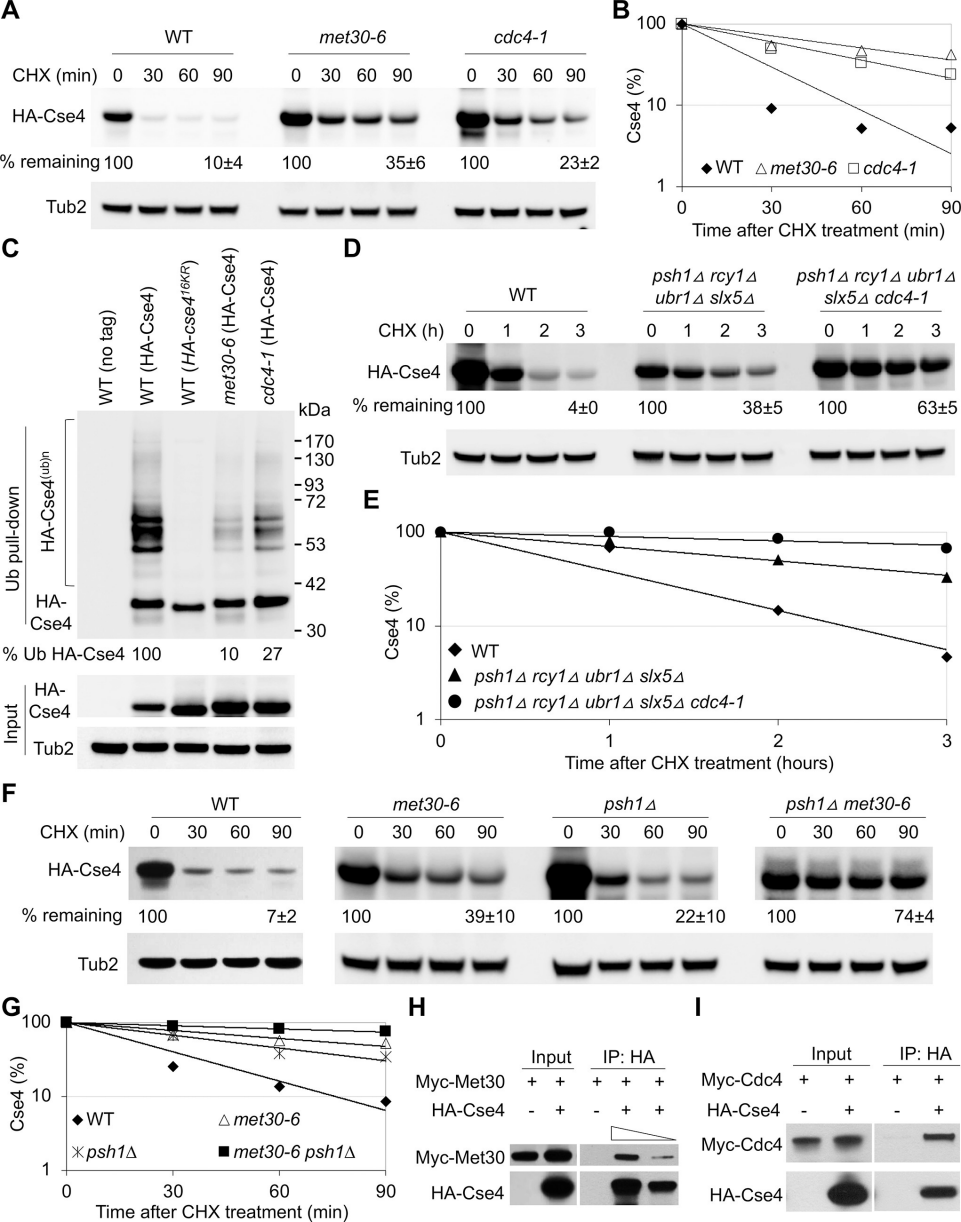
Met30 and Cdc4 interact with Cse4 and regulate ubiquitin-mediated proteolysis of Cse4. (A) Increased stability of overexpressed Cse4 in *met30-6* and *cdc4-1* strains. Western blot analysis was performed with whole cell extracts (WCE) prepared from strains expressing *GAL-HA-CSE4* (pMB1597) grown in galactose media for one hour for WT strain and 3 hours for *met30-6* (TSA848) and *cdc4-1* (TSA878) strains at 25°C and probed with anti-HA (HA-Cse4) and anti-Tub2 antibodies (loading control). Percentage of remaining HA-Cse4 (normalized to Tub2) at the 90 minutes after CHX treatment is shown. Results from three biological repeats are shown as mean ± standard deviation. (B) Line graph for results shown in (A). (C) Reduced levels of ubiquitinated Cse4 in *met30-6* and *cdc4-1* strains. Ub pull-down was performed with WCE prepared after growth of strains in galactose medium for one hour for WT (BY4741) and three hours for *met30-6* (YMB9353), and *cdc4-1* (YMB9571) strains carrying vector or *GAL-HA-CSE4* (pMB1597) at 25°C. WT strains expressing non-tagged Cse4 (empty vector) or HA- cse4^(16KR)^ (pMB1892) were used as negative control for laddering pattern of ubiquitinated Cse4. Western blots were probed with anti-HA antibody. The percentage of ubiquitinated Cse4 is calculated by normalizing the amount of ubiquitinated Cse4 from the Ub pull-down to the levels of non-modified Cse4 in the input where WT is set to 100%. (D) *cdc4-1* increases the stability of overexpressed Cse4 in quadruple mutant (*psh1Δ slx5Δ rcy1Δ ubr1Δ*)(YHR333) strain. The stability of overexpressed Cse4 (pMB1458) was examined in WT, quadruple (YMB11244) and quintuple (*psh1Δ slx5Δ rcy1Δ ubr1Δ cdc4-1*) (YMB11245) mutant strains. Growth in galactose medium was for two hours for WT and quadruple strains and three hours for the quintuple strain. The average of percentage of remaining HA-Cse4 from two biological repeats at 90 min post CHX treatment is shown. (E) Line graph for result shown in (D). (F) *met30-6* further increases the stability of overexpressed Cse4 in *psh1Δ* strain. Stability of overexpressed Cse4 is determined as in (A) for WT(BY4741), *met30-6* (YMB9353), *psh1Δ* (YMB9352) and *met30-6 psh1Δ* (YMB9350) strains. WCE prepared after growth of strains in galactose medium for one hour for WT and *psh1Δ* strains and 3 hours for *met30-6* and *met30-6 psh1Δ* strains. The results represent the average of two biological repeats. A shorter (non-saturated) exposure of Western blot results for *met30-6 psh1Δ* is shown and used for quantification. (G) Line graph for results shown in (F). (H) Cse4 interacts with Met30 *in vivo*. Protein extracts from a WT strain (BY4741) expressing Myc-Met30 (pK699) with either vector (pMB433) or *GAL-HA-CSE4* (pMB1597) were prepared after transient induction of Cse4 in galactose medium for 3 hours at 25°C. Input or IP (anti-HA) samples were analyzed by Western blot and probed with anti-Myc and anti-HA antibodies. For quantification, IP samples in two concentrations (undiluted and diluted 1:3) were loaded (indicated by the triangle). (I) Cse4 interacts with Cdc4 *in vivo*. Protein extracts from *Myc-CDC4* strain (YMB9674) with either vector (pMB433) or *GAL-HA-CSE4* (pMB1597) were prepared after transient induction of Cse4 in galactose medium for 3 hours at 25°C. Input or IP (anti-HA) samples were analyzed by Western blot and probed with anti-Myc and anti-HA antibodies.

Defects in Cse4 proteolysis contribute to *GAL-CSE4*-mediated SDL in *psh1Δ*, *doa1Δ*, *slx5Δ* and *hir2Δ* strains [19, 24–26, 33]. Hence, we examined the stability of overexpressed HA-Cse4 in WT, *met30-6* and *cdc4-1* strains using whole cell extracts from strains grown at the permissive temperature of 25°C. Cse4 was rapidly degraded in the WT strain 90 minutes after cycloheximide (CHX) treatment (Fig 3A and 3B). However, the stability of Cse4 was significantly higher in *met30-6* and *cdc4-1* strains (Fig 3A and 3B). Given that F-box proteins Met30 and Cdc4 function in a complex with Skp1 and Cdc53, these results show that SCF-Met30 and SCF-Cdc4 contribute to the proteolysis of overexpressed Cse4.

To determine whether the higher stability of overexpressed Cse4 in *met3-6* and *cdc4-1* strains is due to defects in ubiquitination, we assayed the levels of ubiquitinated Cse4 of *GAL-HA-CSE4* strain and a non-tagged WT strain as the control by performing an affinity pull-down of ubiquitinated proteins using Ub-binding agarose. As reported previously [19], ubiquitinated Cse4 is detected as a laddering pattern in WT cells expressing HA-Cse4, and no signal was observed in cells without the HA tag (Fig 3C). WT strain overexpressing HA-cse4^16KR^ (non-ubiquitinable Cse4 mutant) did not show laddering pattern but the presence of non-modified Cse4 after Ub pull-down. As reported previously [19], these results confirms that the laddering represents ubiquitinated Cse4, and the non-modified Cse4 is detected due to interaction of Cse4-interacting proteins bound to Ub-binding agarose. Consistent with the possible role of SCF-Met30 and SCF-Cdc4 for Ub-dependent Cse4 proteolysis, the levels of ubiquitinated Cse4 was reduced in the *met30-6* and *cdc4-1* strains (Fig 3C).

Previous studies have shown that overexpressed Cse4 is not completely stabilized in a quadruple mutant for E3 ubiquitin ligase or its co-factor namely *psh1Δ slx5Δ rcy1Δ ubr1Δ* [37]. To assess the contribution of SCF-Met30 and SCF-Cdc4 in Cse4 proteolysis relative to other E3 ligases identified so far, we created quintuple mutants of *cdc4-1* with *psh1Δ slx5Δ rcy1Δ ubr1Δ*. Protein stability assays showed much higher stability of overexpressed Cse4 in the *psh1Δ slx5Δ rcy1Δ ubr1Δ cdc4-1* strain when compared to the quadruple strain (Fig 3D and 3E). We were unable to create a *psh1Δ slx5Δ rcy1Δ ubr1Δ met30-6* strain, and since Psh1 is a major player in proteolysis of overexpressed Cse4 [24, 25], we created a *psh1Δ met30-6* strain to assess epistasis. Protein stability assays showed that Cse4 was more stable in the *psh1Δ met30-6* double mutant strain when compared to the WT and single *met30-6* or *psh1Δ* strains (Fig 3F and 3G). Based on these results, we conclude that SCF-Met30 and SCF-Cdc4 constitute one of the major pathways for proteolysis of Cse4, and SCF-Met30 and SCF-Cdc4 may function independently from Psh1, Slx5, Rcy1 and Ubr1.

F-box proteins interact with their substrates and function as substrate receptors in the context of SCF ligases. We therefore performed co-immunoprecipitation (Co-IP) experiments to examine if Cse4 interacts with Met30 or Cdc4 *in vivo*, using strains expressing Myc-Met30 or Myc-Cdc4 with and without HA-Cse4. Western blot analysis showed that Myc-Met30 (Fig 3H) and Myc-Cdc4 (Fig 3I) co-immunoprecipitated with HA-Cse4. No signal was detected in the untagged strains. Taken together, these results show that Met30 and Cdc4 interact with Cse4 *in vivo* and regulate ubiquitin-mediated proteolysis of overexpressed Cse4.

### SCF-Met30 and SCF-Cdc4 regulate proteolysis of Cse4 under physiological conditions

**Fig 4:**
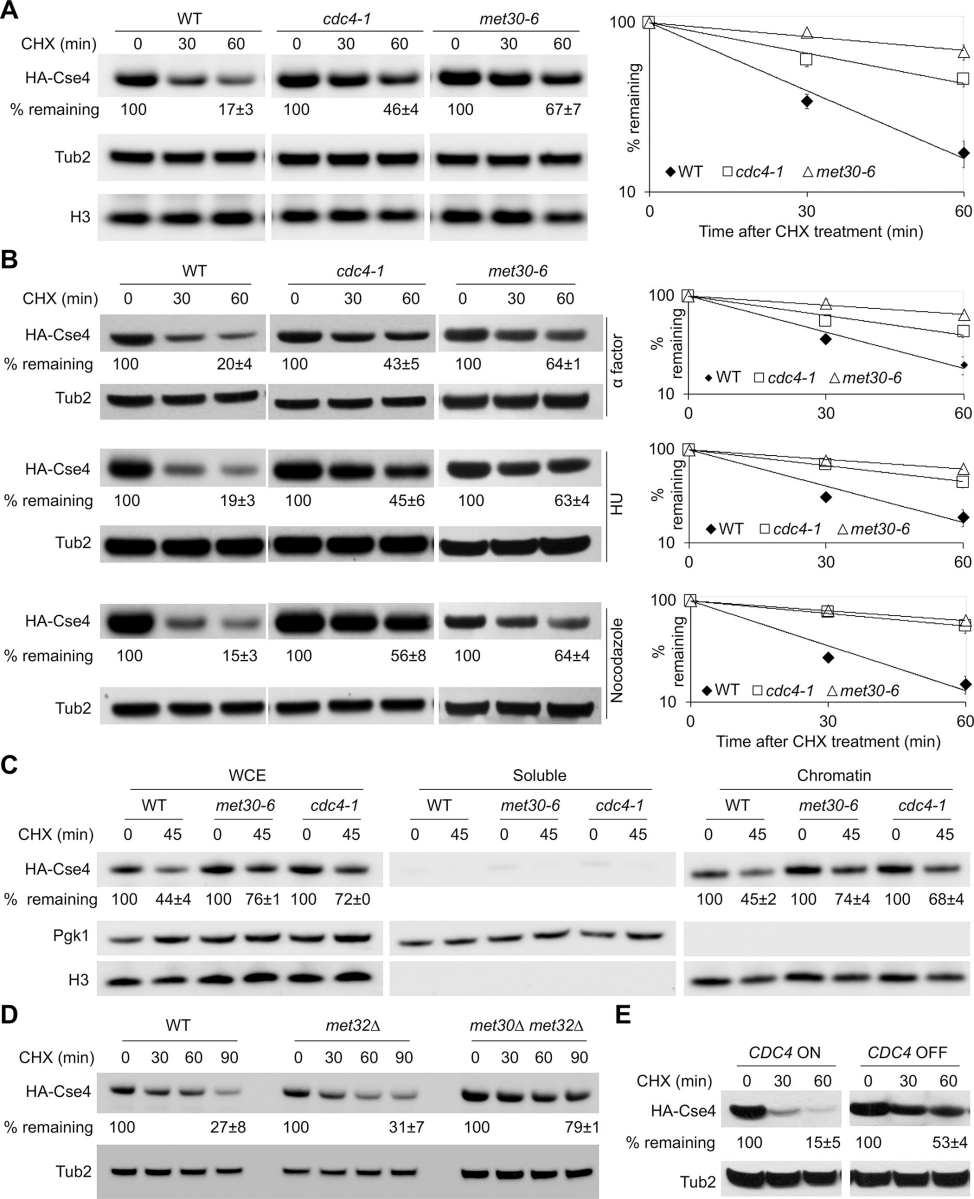
Met30 and Cdc4 regulate stability of endogenous Cse4 independent of cell cycle stage. (A) Increased stability of endogenous HA-Cse4 but not histone H3 in *met30-6* and *cdc4-1* strains. Western blot analysis was performed using WCE from WT (YMB9673), *cdc4-1* (YMB9571), and *met30-6* (YMB8789) strains expressing endogenous HA-Cse4 grown at 25°C. Western blots were probed with anti-HA, anti-H3 and anti-Tub2 (loading control) antibodies. Percentage of remaining HA-Cse4 at 60 minutes after CHX treatment (50 μg/ml) is indicated. Line graphs of the results at different time points is shown on the right. Results from at least two biological experiments are shown as mean ± average deviation. (B) Defects in Cse4 proteolysis in *cdc4-1* and *met30-6* strains are cell cycle independent. Levels of endogenous HA-Cse4 were analyzed by Western blot analysis as described in (A) except WCE were prepared from cells arrested in either G1 phase (with alpha factor), S phase (with hydroxyurea; HU), or G2/M phase (with nocodazole) for 90 min (S2 Fig). Percentage of remaining HA-Cse4 at 60 minutes after CHX treatment (50 μg/ml) is indicated. Line graphs of the results at different time points are shown on the right. Results from two biological experiments are shown as mean ± average deviation. (C) Stabilized Cse4 is enriched in chromatin. Whole cell extracts, soluble and chromatin fractions from WT (YMB9673), *cdc4-1* (YMB9571) and *met30-6* (YMB8789) strains expressing endogenous HA-Cse4 grown at 25°C were analyzed by Western blot analysis using anti-HA (HA-Cse4), anti-Tub2, and anti-H3 antibodies. Tub2 and histone H3 were used as markers for soluble and chromatin fractions, respectively. Percentage of HA-Cse4 remaining after 45 minutes of CHX treatment are shown. Results from two biological experiments are shown as mean ± average deviation. (D) Deletion of *MET32* does not suppress the defect in Cse4 proteolysis in *met30Δ met32Δ s*train. Western blot analysis was performed with WCE from WT (YMB9673), *met32Δ* (YMB10859) and *met30Δ met32Δ* (YMB10799) strains grown at 25°C. Western blots were probed with anti-HA or anti-Tub2 antibodies. Percentage of HA-Cse4 remaining at 90 minutes after CHX treatment (50 μg/ml) is indicated. Results from two biological experiments are shown as mean ± average deviation. (E) Endogenous HA-Cse4 is stabilized upon depletion of Cdc4. The *CDC4* shut-off strain (YMB10212) expressing endogenous HA-Cse4 was grown in galactose at 25°C. CHX (50 ug/ml) treated cells were collected at indicated time points from galactose grown culture (*CDC4*-ON) or after shift to glucose medium (*CDC4*-OFF) for 60 min. Western blots were probed with anti-HA or anti-Tub2 (used as a loading control) antibodies. Percentage of HA-Cse4 remaining at 60 minutes after CHX treatment is indicated. Results of at least two biological experiments are shown as mean ± average deviation.

Our results have shown a role for SCF-Met30 and SCF-Cdc4 in proteolysis of overexpressed Cse4. Degradation of overexpressed proteins is often triggered by unfolded proteins in the overproduced protein pool due to escape from the folding machinery or saturation of the natural site. In order to investigate the physiological role of SCF-Met30 and SCF-Cdc4 in proteolysis of Cse4, we examined the stability of HA-Cse4 expressed from its native promoter at its endogenous locus. Western blot analysis was performed on whole cell extracts prepared from cells grown at the permissive temperature of 25°C, and HA-Cse4 levels were quantified at the indicated time points after CHX treatment. HA-Cse4 was rapidly degraded in WT cells but remained relatively stable in *cdc4-1* and *met30-6* strains at 60 minutes post-CHX treatment (Fig 4A). The stability of histone H3 did not change in *cdc4-1* and *met30-6* strains compared to the WT strain (Fig 4A). Based on these results, we conclude that SCF-Met30 and SCF-Cdc4 regulate proteolysis of endogenous Cse4 under physiological conditions.

We next investigated whether SCF-Met30 and SCF-Cdc4 regulate proteolysis of Cse4 in a cell cycle-dependent manner. Protein stability assays were done using WT, *cdc4-1* and *met30-6* strains arrested in G1 (α factor), S phase (with hydroxyurea; HU) and M phase (with nocodazole) at 25°C. We performed Fluorescence Activated Cell Sorting (FACS) and nuclear morphology analysis to determine the cell cycle arrest for each strain (S2 Fig). Consistent with previous observations [24], Cse4 is rapidly degraded in the WT cells in G1, S and M phases of the cell cycle (Fig 4B). However, independent of cell cycle arrest condition, Cse4 was stabilized in *cdc4-1* and *met30-6* strains, indicating that SCF-Met30 and SCF-Cdc4 are required for Cse4 degradation independent of specific cell cycle stages.

To determine if the higher levels of Cse4 in whole cell extracts of *met30-6* and *cdc4-1* strains are due to higher levels of Cse4 in the soluble or chromatin fraction, we performed subcellular fractionation of endogenous Cse4 in WT, *cdc4-1* and *met30-6* strains with or without CHX. Our results showed that chromatin-associated Cse4 was more stable in the *met30-6* and *cdc4-1* strains when compared to the WT strain (Fig 4C). Consistent with previous results [26], Cse4 was barely detectable in the soluble fraction of WT, *met30-6* and *cdc4-1* strains (Fig 4C). Taken together, these results suggest that SCF-Met30 and SCF-Cdc4 restrict the level of chromatin -bound Cse4.

To examine if the defects in Cse4 proteolysis in *met30-6* were allele-specific, we made use of the observation that the essentiality of *MET30* is suppressed by a deletion of *MET32* [48]. The stability of Cse4 was examined in a *met30Δ met32Δ* strain. As expected, *met30Δ met32Δ* is viable and does not exhibit temperature sensitivity for growth (S3B Fig). We observed defects in Cse4 proteolysis in *met30Δ met32Δ* when compared to WT or a *met32Δ* strain at 90 minutes post-CHX treatment (Fig 4D). In a second approach, we created an auxin-inducible Met30 degron system (AID-MET30) to deplete Met30 in the presence of TIR1 and auxin [63, 64]. Defects in Cse4 proteolysis upon depletion of Met30 were observed after 2 hours of auxin treatment in cells expressing *TIR1* but not in cells without auxin treatment or strains lacking *TIR1* at 90 min after CHX treatment (S3A Fig).

We next tested if defects in Cse4 proteolysis were due to loss of Cdc4 activity rather than hypermorphic effects of the *cdc4-1* allele. We created an auxin-inducible degron system targeting Cdc4, however, we failed to see a significant depletion of Cdc4 upon auxin treatment. Hence, we created a Cdc4-shut off strain in which *CDC4* is expressed from a *GAL1* promoter at the *CDC4* endogenous locus. In this strain, *CDC4* was overexpressed in galactose-containing medium and depleted upon growth in glucose medium for 60 minutes (S3C Fig). Defects in Cse4 proteolysis were observed after depletion of Cdc4 (*CDC4* OFF) when compared to the control *CDC4*-ON strain at 60 minutes post-CHX treatment (Fig 4E). Taken together, these results show that defects in Cse4 proteolysis are not specific to *met30-6* and *cdc4-1* mutant alleles and define a role for SCF-Met30 and SCF-Cdc4 in proteolysis of endogenous Cse4 under physiological conditions.

### Met30 and Cdc4 interact *in vivo* and cooperatively regulate the proteolysis of Cse4

**Fig 5:**
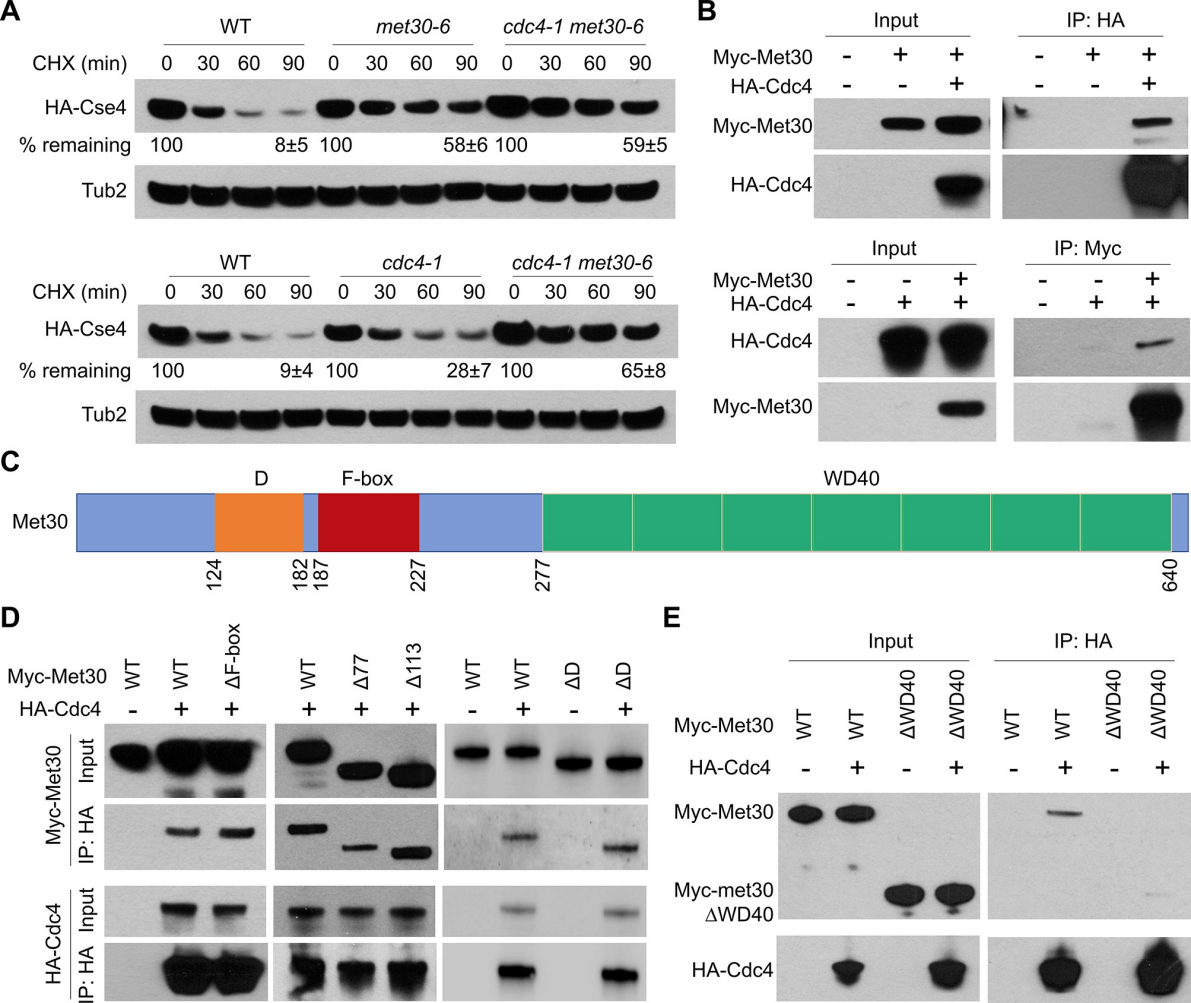
Met30 and Cdc4 interact *in vivo* and cooperatively regulate proteolysis of Cse4. (A) Cdc4 and Met30 cooperatively regulate proteolysis of Cse4. Western blot analysis was performed with WCE prepared from WT (YMB9673), *met30-6* (YMB8789), *cdc4*-*1* (YMB9571) and *cdc4-1 met30-1* (YMB10033) strains expressing endogenous HA-Cse4. The percentage of remaining HA-Cse4 at 90 minutes after CHX treatment (50 μg/ml) is indicated. Results from two biological experiments are shown as mean ± average deviation. (B) Met30 interacts with Cdc4 *in vivo*. Top panel: Co-IP was performed with anti-HA antibody using WCE from *cdc4Δ*::*HA-CDC4* strain (YMB10217) with *Myc-MET30* (pK699); control strains WT (BY4741) with either vector (pRS415) or *Myc-MET30* (pK699) grown in selective glucose medium at 25°C. Western blot analysis of Input and IP (anti-HA) samples were analyzed using anti-HA and anti-Myc antibodies. Bottom Panel: Co-IP was performed with anti-Myc using WCE from *cdc4Δ*::*HA-CDC4* strain (YMB10217) with *Myc-MET30* (pK699); and control strains (YMB10217) with vector (pRS415) grown at 25°C. Western blot analysis of Input and IP (anti-HA) samples were analyzed using anti-HA and anti-Myc antibodies. All tagged proteins are expressed from their native promoter. (C) Schematic of Met30 domains. Homodimerization domain (D), F-box and WD40 domain with amino acid numbers indicated. (D) The N-terminus, homodimerization domain (D domain) and F-box of Met30 are dispensable for the interaction of Met30 and Cdc4. Co-IP experiments were performed with anti-HA using WCE from a *cdc4Δ*::*HA-CDC4* strain (YMB10217) with *Myc-MET30* (pK699), *Myc-met30ΔF-box* (*Δ*187–227 aa, pK680), *Myc-met30Δ77* (*Δ*1–77 aa, pMB1835), *Myc-met30Δ113* (*Δ*1–113 aa, pMB1837) or *Myc-met30ΔD* (*Δ*124–182 aa, pMB1830) and control WT strain (BY4741) with *Myc-MET30* (pK699) or *Myc-met30ΔD* (*Δ*124–182 aa, pMB1830) grown at 25°C. Western blot analysis of Input and IP (anti-HA) samples were probed with anti-Myc and anti-HA antibodies. All tagged proteins are expressed from their native promoters. (E) The WD40 domain of Met30 is required for the interaction of Met30 and Cdc4. Co-IP experiments were performed with anti-HA using WCE from a *cdc4Δ*::*HA-CDC4* strain (YMB10217) with *Myc-MET30* (pK699) or *Myc-met30ΔWD40* (*Δ*277–640 aa, pMB1861) and control WT strain (BY4741) with *Myc-MET30* (pK699) or *Myc-met30ΔWD40* (*Δ*277–640 aa, pMB1861) grown at 25°C. Western blot analysis of Input and IP (anti-HA) samples were analyzed using anti-HA and anti-Myc antibodies. All tagged proteins are expressed from their native promoters.

Met30 and Cdc4 have a high degree of homology (53.7% amino acid sequence similarity) and both proteins interact with Cse4 to regulate proteolysis of Cse4 (Fig 3H and 3I and Fig 4A). Our results prompted us to investigate the contribution and functional relationship between Cdc4 and Met30 in Cse4 proteolysis. The stability of endogenous Cse4 was examined in *cdc4-1*, *met30-6* and *cdc4-1 met30-6* double mutant strains grown at the permissive temperature of 25°C. As shown earlier (Fig 4A), Western blot analysis of whole cell extracts showed higher stability of Cse4 in *met30-6* and *cdc4-1* strains when compared to WT strains 90 minutes after CHX treatment (Fig 5A). The stability of Cse4 in the *cdc4-1 met30-6* double mutant strain was not significantly higher than that observed in the *met30-6* strain, suggesting that Met30 and Cdc4 may function in the same pathway to regulate Cse4 proteolysis.

To determine if Met30 and Cdc4 physically interact *in vivo*, a Co-IP was performed with strains expressing Myc-Met30 and HA-Cdc4 from their endogenous promoters. Myc-Met30 was detected in an IP with HA-Cdc4, but not in the control strain without HA-Cdc4 (Fig 5B, Top). Likewise, HA-Cdc4 was detected in an IP with Myc-Met30 but not in the control strain lacking Myc-Met30 (Fig 5B, Bottom). These results provide evidence for an *in vivo* interaction between Met30 and Cdc4 under normal physiological conditions.

Several functional domains have been identified in Met30. The most important are the F-box for interaction with Skp1, the D-domain for homodimerization and the WD40 domain for substrate recognition (Fig 5C). Homodimerization of SCF complexes mediated by the D-domain is important for their function [65–67]. We sought to identify the domain(s) of Met30 that are responsible for Cdc4 interaction using Co-IP experiments, expecting that the D-domain would mediate Met30/Cdc4 binding. We used *met30* mutants with deletions of the N-terminus (Δ77 and Δ113), F-box (ΔF), D-domain (ΔD) or WD40 (ΔWD40) domain. Our results showed that deletions of the F box, the N-terminus and, to our surprise, the D-domain of Met30 do not abolish the interaction between Met30 with Cdc4 (Fig 5D). However, met30ΔWD40 shows reduced Cdc4 interaction (Fig 5E). Note that the Cdc4/Met30 binding is independent of the Met30 F-box, indicating that other SCF components are not involved in the interaction. Taken together, these results show that the WD40 domain of Met30, but not the F-box, N-terminus or D-domain, is required for the interaction of Met30 with Cdc4.

### Met30 regulates the interaction of Cdc4 with Cse4

**Fig 6:**
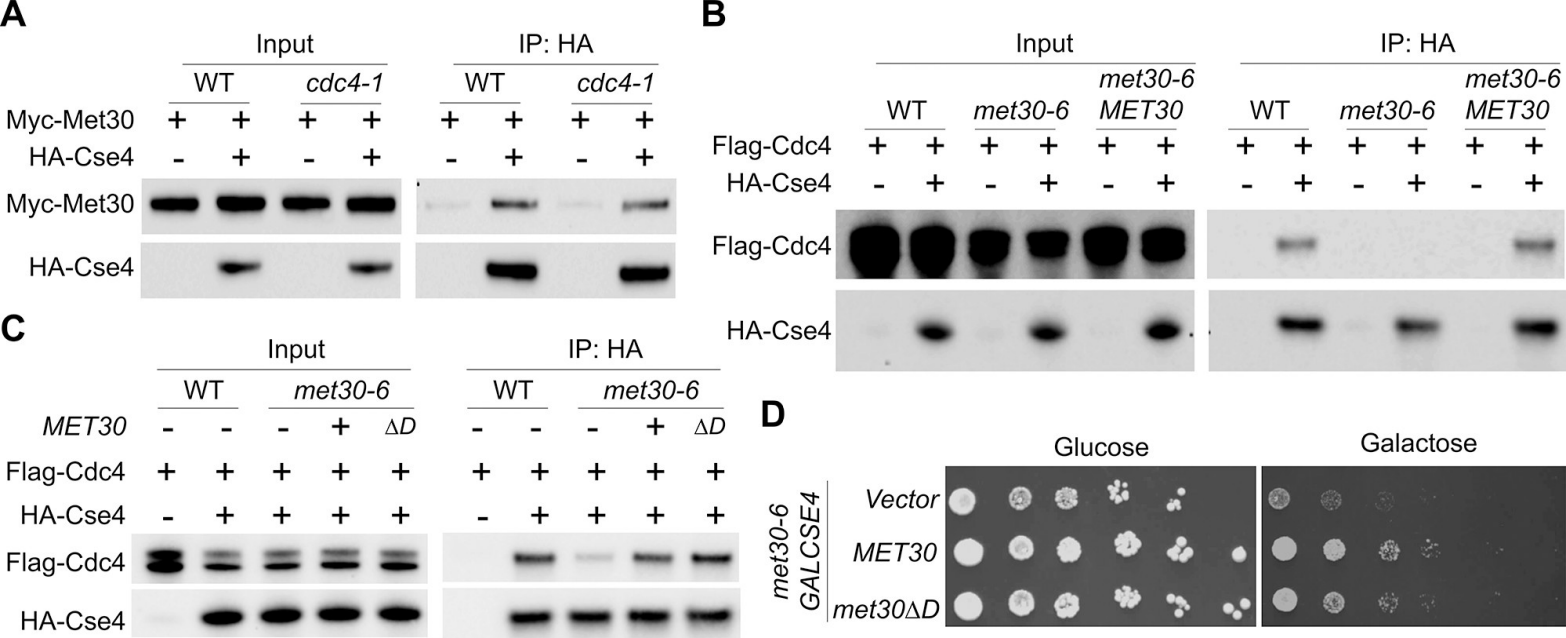
Met30 regulates the interaction of Cdc4 with Cse4. (A) The interaction between Met30 and Cse4 is not affected in a *cdc4-1* strain. Co-IP experiments were performed with anti-HA using WCE from WT strain (YMB9673) expressing *Myc-MET30* (pK699) with vector (pRS426) or *HA-CSE4* (pMB1831) and *cdc4-1* (YMB9571) cells expressing *Myc-MET30* (pK699) with vector (pRS426) or *HA-CSE4* (pMB1831) grown in selective glucose medium at 25°C. Input and IP (anti-HA) samples were analyzed by Western blot analysis and probed with anti-Myc and anti-HA antibodies. All tagged proteins are expressed from their native promoter. (B) The interaction between Cdc4 and Cse4 is reduced in the *met30-6* strain. Co-IP experiments were performed with anti-HA using WCE from a control WT strain (YMB9673) expressing *Flag-CDC4* (pMB1840) with vector (pRS426) or *HA-CSE4* (pMB1831) and *met30-6* strain (YMB8789) expressing *Flag-CDC4* (pMB1840) with vector (pRS426) or *HA-CSE4* (pMB1831). To check the complementation effects on Cdc4/Cse4 interaction, Co-IP experiments were performed with anti-HA using WCE from *met30-6* cells (YMB8789) expressing *MET30* (pK699) and *Flag-CDC4* (pMB1840) with vector (pRS426) or *HA-CSE4* (pMB1831) grown in selective glucose medium at 25°C. Input and IP (anti-HA) samples were analyzed by Western blot analysis and probed with anti-Flag and anti-HA antibodies. All tagged proteins are expressed from their native promoter. (C) The homodimerization domain of Met30 is dispensable for the interaction of Cdc4 with Cse4. Co-IP experiments were performed with anti-HA using WCE from control WT strain (YMB9673) expressing *Flag-CDC4* (pMB1840) with vector (pRS426) or *HA-CSE4* (pMB1831). To check for the complementation of defects in interaction between Cdc4 and Cse4, Co-IP experiments were performed with anti-HA using WCE from *met30-6* strain (YMB8789) expressing *Flag-CDC4* (pMB1840) and *HA-CSE4* (pMB1831) with vector (pRS413), *MET30* (pK699) or *met30*Δ*D* (pMB1951). Input and IP (anti-HA) samples were analyzed by Western blot analysis and probed with anti-Flag and anti-HA antibodies. (D) The homodimerization domain of Met30 is dispensable for the *GAL-CSE4*-mediated lethality in a *met30-6* strain. *met30-6* (YMB8789) with pMB1807 (*GAL-CSE4*) was transformed with Vector (pRS416), *MET30* (pP88) or *met30*Δ*D* (pMB1918) on a *CEN* plasmid. Strains were grown to logarithmic phase and five-fold serial dilutions were plated on either glucose- or galactose-containing plates and incubated at 25°C for 5–6 days.

To investigate the possible cooperative role of Met30 and Cdc4 in Cse4 proteolysis, we examined the interdependency of Met30 and Cdc4 for their interaction with Cse4. Co-IP experiments showed that the interaction between Myc-Met30 and HA-Cse4 was not affected in the *cdc4-1* strain (Fig 6A). However, the interaction of Flag-Cdc4 with HA-Cse4 was greatly reduced in the *met30-6* strain (Fig 6B). As expected, Flag-Cdc4 showed an interaction with HA-Cse4 in wild type cells. We determined that the defect in the interaction of Cdc4 with Cse4 is linked to *met30-6*, as plasmid-borne *MET30* restored the interaction of Flag-Cdc4 and HA-Cse4 in the *met30-6* strain (Fig 6B). These results suggest that the interaction of Cdc4 with Cse4 is mediated by Met30, a conclusion supported further by the lack of Cdc4 binding to Cse4 in a *met30*Δ *met32*Δ strain (S4A Fig).

Previous studies have shown that homodimerization of SCF complexes is important for their function [65–67]. The unexpected result that the D-domain of Met30 is dispensable for the interaction of Met30 with Cdc4 (Fig 5D) [66, 68] suggesting that the D-domain of Met30, albeit essential for viability, may not be required for the interaction of Cdc4 with Cse4. Hence, we examined if *met30*Δ*D* can suppress the binding defect of Cdc4 with Cse4. As reported previously [66], *met30*Δ*D* failed to suppress the temperature sensitive growth of the *met30-6* strain (S4B Fig) or rescue the defective ubiquitination of Met4 in a *met30*Δ *met32*Δ strain (S4C Fig). However, consistent with our hypothesis, Co-IP experiments showed that *met30*Δ*D* can mediate the interaction of Cdc4 with Cse4 (Fig 6C). We therefore asked whether *met30*Δ*D* can overcome *GAL-CSE4* mediated SDL in the *met30-6* strain. Indeed, *met30*Δ*D* suppresses the *GAL-CSE4* SDL in *met30-6* strain (Fig 6D), indicating that the homodimerization domain of Met30 is neither required for the interaction of Cdc4 with Met30 or Cse4, nor for suppression of SDL due to Cse4 overexpression. Interestingly, the requirement of both Cdc4 and Met30 for ubiquitination seems to be Cse4-specific since Met4, a SCF-Met30 substrate, does not exhibit defects in ubiquitination in a *cdc4-1* strain (S5 Fig). Together, our results suggest that Met30 directs the interaction of Cdc4 with Cse4 and that Cdc4 participates in the complex through its interaction with the WD40 domain of Met30.

### SCF-Met30 and SCF-Cdc4 prevent mislocalization of Cse4 to non-centromeric regions and maintain chromosomal stability

**Fig 7:**
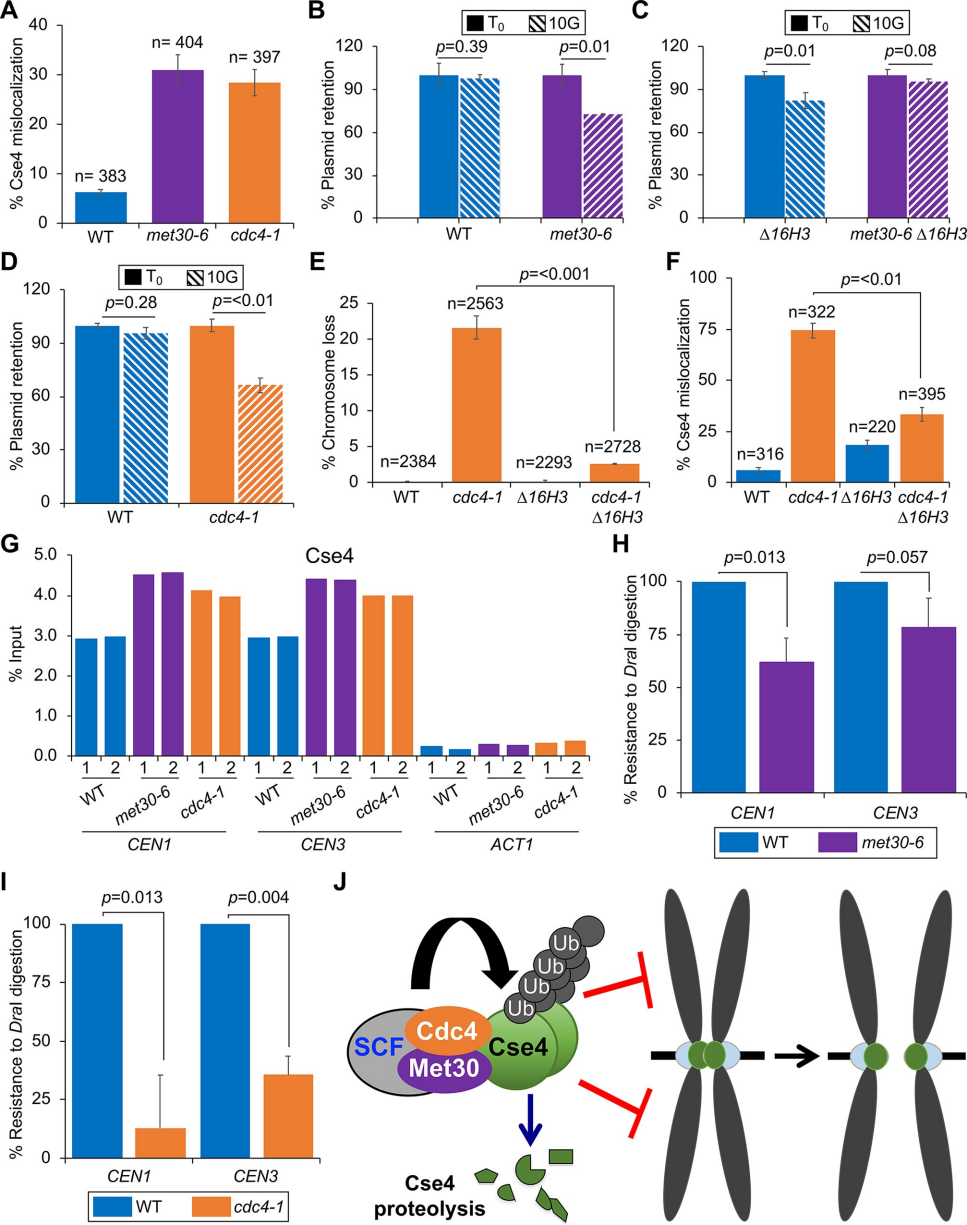
Mislocalization of Cse4 contributes to defects in chromosome segregation in *met30-6* and *cdc4-1* strains. (A) Endogenous Cse4 is mislocalized to non-centromeric regions in *met30-6* and *cdc4-1*strains. Localization of Cse4 was examined by chromosome spreads in WT (YMB8788), *met30-6* (YMB8789) and *cdc4-1* (YMB9571) strains grown at 25°C. Cse4 localization was determined using Cy3-conjugated secondary antibody and DNA was stained with DAPI. Cse4 localization is restricted to 1–2 foci was scored as normal, mislocalization of Cse4 results in more than 3 foci or increased area of Cse4 localization within the nucleus (S6 Fig). Images were acquired with 63X objective with the same exposure time. Error bars represent the standard deviation of three biological experiments. n = number of cells scored. (B) Increased plasmid loss in *met30-6* strain. WT (YMB9983) and *met30-6* (YMB9984) strains transformed with *CEN* plasmid (pRS416) were grown in medium selective (SC-Ura) for the plasmid (denoted as T_0_) and then grown in non-selective medium (YPD) for 10 generations (10G). Equal number of cells from T_0_ and 10G were plated on YPD and SC-Ura plates at 25°C. Plasmid retention was measured by the ratio of colonies grown on SC-URA/YPD. The percentage of plasmid retention (SC-URA/YPD) at 10G is normalized to that at T_0_ where the percentage of plasmid retention is set to 100%. Error bars represent the standard deviation of three biological experiments. (C) Plasmid loss phenotype of *met30-6* strains is suppressed by constitutive expression of histone H3 (Δ*16H3*). WT (YMB9985) and *met30-6* (YMB9986) strains containing Δ*16H*3 were transformed with pRS416 and assayed for plasmid retention as described in (B) above. (D) Increased plasmid loss in *cdc4-1* strain. Plasmid loss was determined as described in (B) with WT (BY4741) and *cdc4-1* (TSA878) strains transformed with pRS416 plasmid. (E) Increased chromosome loss in *cdc4-1* is suppressed by constitutive expression of histone H3 (Δ*16H3*). Loss of the reporter chromosome (RC) was measured using a colony color assay in which loss of the RC results in red sectors in an otherwise white colony. Log phase WT (YPH1015), *cdc4-1* (YMB10365), Δ*16H3* (YMB6331) and *cdc4-1* Δ*16H3* (YMB10366) strains grown in selective medium to maintain the RC, and then plated on complete synthetic medium with limiting adenine at 33°C to allow the loss of the RC. The frequency of chromosome loss was measured by the percentage of colonies that show loss of the RC in the first cell division resulting in a colony which is at least half-red. Three individual isolates were examined for each strain. The results show the average of three biological experiments. Error bars represent standard deviation. n: number of colonies examined. (F) Mislocalization of Cse4 is suppressed by constitutive expression of histone H3 (Δ*16H3)* in a *cdc4-1* strain. Localization of endogenous HA-Cse4 was examined by chromosome spreads as in (A) using WT (YMB10436), *cdc4-1* (YMB10437), Δ*16H3* (YMB10438) and *cdc4-1* Δ*16H3* (YMB10439) strains expressing endogenous *HA-CSE4* at 33°C. n = number of cells scored. (G) The *CEN* levels of Cse4 are not reduced in *met30-6* and *cdc4-1* strains. Wild type (WT, YMB9673), *met30-6* (YMB8789) and *cdc4-1* (YMB9571) strains expressing HA-Cse4 from its native promoter were grown in YPD at 25°C to the logarithmic phase. ChIP for HA-Cse4 was performed using anti-HA agarose beads (A2095, Sigma Aldrich. Enrichment of Cse4 at *CEN1*, *CEN3* and *ACT1* (negative control) was determined by qPCR and is shown as % input. Results of two biological replicates denoted as 1 and 2 are shown. (H) Defects in kinetochore integrity in *met30-6* strains. Nuclei prepared from WT (YMB9673), and *met30-6* (YMB8789) grown to logarithmic phase of growth at 25°C were treated with or without *Dra1*. The extent of *Dra1*-specific cleavage at *CEN1* (302 bp, OMB426/427) and *CEN3* (243bp, OMB244/245) loci was measured by qPCR using equal amount of genomic DNA (100 ng) from these strains. % *Dra1* resistance was quantified as the ratio of *CEN* from uncut /cut samples normalized to that observed in WT set to 100%. Values represent mean±standard deviation of three biological repeats. (I) Defects in kinetochore integrity in *cdc4-1* strains. Assays as described in (H) were done using nuclei prepared from WT (YMB9673), and *cdc4-1* (YMB9571) grown at 33°C. (J) Schematic Model depicting a cooperative role of SCF-Met30 and SCF-Cdc4 in preventing the mislocalization of Cse4 for chromosomal stability. We propose that the interaction of a heterodimer of SCF-Met30/Cdc4 with Cse4 regulates ubiquitin-mediated proteolysis of Cse4, and this prevents stable maintenance of Cse4 at non-centromeric regions for faithful chromosome segregation.

We investigated the physiological consequences of defects in Cse4 proteolysis in *met30-6* and *cdc4-1* strains. Increased stability of overexpressed Cse4 in *psh1*Δ, *slx5*Δ and *hir2*Δ strains correlates with its mislocalization to non-centromeric regions [24–26, 33]. We reasoned that the strong defects in proteolysis of endogenous Cse4 may contribute to its mislocalization and CIN in *met30-6* and *cdc4-1* strains. To examine the localization of Cse4, we performed chromosome spreads, a technique that removes soluble material to allow visualization of chromatin bound Cse4 expressed from its own promoter. Immunofluorescence staining of HA-Cse4 showed one to two discrete Cse4 foci coincident with DAPI (DNA) signal in the majority of WT cells, mislocalization of Cse4 was defined as cells showing more than two Cse4 foci or diffused chromatin-associated Cse4 signal (S6 Fig). Our results showed that the percentage of *met30-6* or *cdc4-1* cells exhibiting Cse4 mislocalization were about four-fold higher when compared to the WT strain (Fig 7A).

Mislocalization of CENP-A and its homologs contributes to CIN in yeast, fly and human cells [4, 6, 7]. To determine if mislocalization of Cse4 in *met30-6* strains contributes to CIN, we tested the ability of cells to retain a centromere-containing plasmid (pRS416 *URA3)* after growth in non-selective medium at the permissive temperature of 25°C. Plasmid retention was measured as the ratio of the number of colonies grown on SC-Ura versus YPD medium. Plasmid retention after 10 generations (10G) of non-selective growth was 98.8% for a WT strain compared to 72.7% for the *met30-6* strain (*p*-value = 0.01, Fig 7B). We confirmed that the reduced plasmid retention in *met30-6* is directly linked to the mutant allele because *met30-6* expressing WT *MET30* showed higher plasmid retention than *met30-6* strain (S7A Fig). Deletion of *MET32* suppresses the temperature sensitivity of *met30-6* strains [47, 49]. Hence, we examined if plasmid retention was higher in a *met30*Δ *met32*Δ strain. Our results showed that the plasmid retention of *met30*Δ *met32*Δ strain remained defective when compared to the WT strain (p value = 0.0017) and was not significantly different from that observed in the *met30-6* strain (S7B Fig). These findings are consistent with our results showing that deletion of *MET32* does not suppress the SDL of *GAL-CSE4 met30-6* (Fig 2D) or defects in Cse4 proteolysis in *met30-6* strain (Fig 4D).

To link the plasmid loss phenotype of the *met30-6* strain to mislocalization of Cse4, we examined the effect of constitutive expression of histone H3 (Δ*16H3*). We previously showed that mislocalization and chromosome segregation defects due to overexpression of the stable cse4 mutant, *cse4*^*16KR*^ (in which all 16 lysines in Cse4 are mutated to arginines), is suppressed by the constitutive expression of histone H3 (Δ*16H3*) [4]. Consistent with the observed increase in plasmid loss due to Cse4 mislocalization, high levels of plasmid retention (95.4%) were observed in the *met30-6* strain expressing Δ*16H3* at 10G (*p*-value = 0.08, Fig 7C), this is despite the fact that, in agreement with previous results showing an effect of altered histone stoichiometry on chromosomal stability in WT budding yeast [4] and fission yeast [69], a WT strain containing Δ*16H3* showed reduced plasmid retention (Fig 7C).

We next determined if the SDL of *GAL-CSE4* and stability of endogenously expressed Cse4 in *met30-6* is suppressed by Δ*16H3*. Our results showed that Δ*16H3* partially suppressed the SDL of *GAL-CSE4* (S8A Fig) and reduced the stability of endogenous Cse4 and its enrichment in chromatin in *met30-6* (S8B Fig). Furthermore, growth assays showed that Δ*16H3* failed to suppress the temperature sensitive growth defect of *met30-6* strain at 35°C and 37°C (S8C Fig), suggesting the suppression of *GALCSE4* SDL and proteolysis defects in *met30-6* by Δ*16H3* is specific for Cse4. Taken together, these results show that defects in Cse4 proteolysis, and mislocalization contribute to increased plasmid loss in *met3-6* strain.

We next examined the role of Cdc4 in preventing mislocalization of endogenous Cse4 to maintain chromosomal stability. The plasmid retention rate after 10 generations of non-selective growth (10G) in the *cdc4-1* strain (66%) was significantly lower than the WT strain (96%) at 25°C (Fig 7D). We confirmed that the plasmid loss phenotypes in *cdc4-1* is linked to the mutant allele because the plasmid loss rate in *cdc4-1* expressing the WT *CDC4* was reduced when compared with that in *cdc4-1* strains (S7A Fig). To further validate the role of Cdc4 in chromosomal stability, we used an independent colony sectoring assay and determined the frequency of loss of a reporter chromosome (RC) [70]. Loss of the RC leads to red sectors in an otherwise white colony. The metabolic defects in the *met30-6* strain did not allow us to distinguish the loss of the RC and we could therefore not utilize this assay for *met30-6* strains. The *cdc4-1* strain did not show higher loss of RC at 25°C, but showed a significantly higher loss of the RC when compared to the WT strain at 33°C (22% vs 0.17%, Fig 7E). Protein stability assays confirmed higher levels of endogenous Cse4 in the *cdc4-1* strain than the WT strain at 33°C (S9 Fig). As observed for *met30-6* strains (Fig 7C), Δ*16H3* suppressed the chromosome loss in the *cdc4-1* strain (2.6% vs 22%, Fig 7E). We hypothesized that the Δ*16H3*-mediated suppression of chromosome loss in the *cdc4-1* strain is due to reduced Cse4 mislocalization. Chromosome spreads were used to examine the localization of Cse4 in WT and *cdc4-1* strains with or without Δ*16H3*. Higher levels of Cse4 mislocalization were observed in the *cdc4-1* strain but not in the WT strain (74% vs 5.6%) (Fig 7F). We determined that Δ*16H3* suppressed the mislocalization of Cse4 in the *cdc4-1* strain (33% vs 74%). These observations support our hypothesis that Δ*16H3*-mediated suppression of chromosome loss in the *cdc4-1* strain is due to reduced Cse4 mislocalization. Taken together, these results show that both SCF-Met30 and SCF-Cdc4 are required to prevent mislocalization of Cse4 for maintaining chromosomal stability.

We next performed genome-wide ChIP-seq experiments to examine the localization of Cse4 using WT, *met30-6* and *cdc4-1* strains expressing HA-Cse4 from its own promoter, or a control strain with untagged Cse4. In control experiments, as expected, enrichment of HA-Cse4 at *CENs* was only observed in WT strain with HA-Cse4, in contrast no significant peaks at non-centromeric regions or *CENs* were detected in WT strain with untagged Cse4 (S10A, S10B, S10C and S10D Fig). ChIP-seq experiments showed an enrichment of HA-Cse4 at *CENs* in the WT, *met30-6* and *cdc4-1* strains (S11A and 11B Fig). The levels of Cse4 at the *CEN* were higher in *met30-6* (p value <0.001) and *cdc4-1* (p value <0.01) strains than the WT strain (S11C Fig). ChIP-qPCR confirmed that the levels of Cse4 at the *CEN* were higher in *met30-6* and *cdc4-1* strains than the WT strain (Fig 7G). Though non-*CEN* peaks of Cse4 are detected in *met30-6* and *cdc4-1* strains, peak enrichment (vs. the 10-kb local background) is much lower than that observed for any of the 16 *CENs*, and not statistically different from that observed in WT cells (S11D Fig). Thus, the extracentromeric localization of Cse4 observed in *met30-6* and *cdc4-1* strains by chromosome spread cannot be attributed to highly increased accumulation of Cse4 at discrete, non-centromeric loci; rather, we conclude that endogenously expressed Cse4 in *met30-6* and *cdc4-1* strains accumulates at marginally increased levels throughout the genome.

Overexpression of CENP-A contributes to the mislocalization of the CENP-A interacting protein CENP-C (Mif2 in yeast) and CIN in human cells [6], so we examined if Mif2 is also mislocalized in *met30-6* and *cdc4-1* strains. Chromosome spread experiments showed that Mif2 was localized to one or two foci in WT cells. Mislocalization of Mif2 was barely detectable in cells that do not show mislocalization of Cse4 (S12A Fig). However, the number of cells that showed mislocalization of both Cse4 and Mif2 was significantly higher in *cdc4-1* and *met30-6* strains (S12A Fig). ChIP-qPCR showed that the *CEN* association of Mif2 was similar in the WT, *met30-6* and *cdc4-1* strains (S12B Fig). To exclude the possibility that mislocalization of Cse4 or Mif2 was due to a kinetochore clustering defect, we examined the localization of a GFP-tagged kinetochore protein, Mtw1-GFP. One or two discrete Mtw1-GFP foci were observed in 95–97% of WT, *met30-6* and *cdc4-1* cells (S12C Fig). Taken together, these results show that Cse4 and Mif2 are mislocalized to non-centromeric regions in *met30-6* and *cdc4-1* strains.

We have recently shown that the CIN phenotype due to mislocalization of CENP-A and CENP-C to non-centromeric regions in human cells results from the weakening of the native kinetochore [6]. To determine if mislocalization of Cse4 contributes to defects in the integrity of the kinetochore in *met30-6* and *cdc4-1* strains, we examined the susceptibility of centromeric (*CEN*) chromatin to digestion by the restriction enzyme *DraI*. There are three closely spaced *DraI* recognition sequences within the *CDE-II* region of budding yeast *CENs* and these are protected from endonuclease digestion due to the kinetochore protein complex [71, 72]. Yeast nuclei were treated with *DraI* endonuclease and *CEN* chromatin was assayed for sensitivity to *DraI* by quantitative PCR using primers flanking *CEN1* and *CEN3*. Similar to the increased *DraI* sensitivity observed previously in kinetochore mutants [34, 59, 71–73], *CEN1* and *CEN3* chromatin in *met30-6* (Fig 7H) and *cdc4-1* (Fig 7I) strains were more susceptible to *DraI* digestion than that observed for a WT strain. We propose that Met30 and Cdc4 act cooperatively to prevent mislocalization of Cse4 and weakening of kinetochores to promote chromosomal stability.

## Discussion

Mislocalization of overexpressed CENP-A and its homologs contributes to aneuploidy in yeast, fly and human cells [4–8]. Over the past few years, several E3 ligases (Psh1, Slx5, Ubr1 and SCF-Rcy1) that can prevent mislocalization of overexpressed Cse4 were identified in yeast [24–27], however, Cse4 is still degraded, albeit less efficiently, in a *psh1*Δ *rcy1*Δ *slx5*Δ *ubr1*Δ quadruple mutant strain [37]. Hence, additional pathways are likely to regulate homeostasis of Cse4 under unperturbed conditions and restrict the localization of Cse4 to centromeric regions for chromosomal stability. Using budding yeast, we provide the first comprehensive analysis of essential genes that are required for growth when Cse4 is overexpressed. Amongst the significant hits of the screen were genes that regulate Ub-proteasome pathways including those encoding components of the SCF complex and its two essential substrate receptors Met30 and Cdc4. We focused our investigation on the role of SCF-Met30 and SCF-Cdc4 and determined that Met30 and Cdc4 interact with and cooperatively regulate ubiquitin-mediated proteolysis of Cse4. Moreover, Met30 regulates the interaction of Cdc4 with Cse4, and defects in proteolysis of Cse4 in *met30-6* and *cdc4-1* mutants lead to mislocalization of Cse4 and increased chromosome loss under normal physiological conditions. In summary, the SCF-Met30/Cdc4 defines a major pathway that regulates cellular levels of Cse4 and prevents its stable maintenance at non-centromeric regions for chromosomal stability.

Our results provide the first evidence for a role of the two essential nuclear F-box/WD40 proteins Met30 and Cdc4 in the ubiquitin-mediated proteolysis of a new substrate, Cse4. Because Met30 or Cdc4 inactivation leads to cell cycle arrest, we carefully considered indirect consequences of cell cycle effects on Cse4 stability. Previous studies have shown that the stability of endogenous Cse4 is independent of the cell cycle in a WT strain [25]. Consistent with a direct role for Met30 and Cdc4 in Cse4 degradation, Cse4 stabilization was observed to a similar extent in *met30-6* and *cdc4-1* mutants arrested in either G1, S or M phases of the cell cycle. In addition, experiments were conducted at the permissive temperature, which allows normal cell cycle progression, confirming that Cse4 stabilization in these mutants is independent from their roles in cell cycle progression. In agreement with these findings, deletion of *MET32* or *SIC1*, which are responsible for cell cycle arrest in *met30* and *cdc4* mutants, respectively [48, 50], do not suppress sensitivity to Cse4 overexpression. Importantly, defects in Cse4 proteolysis are not limited to the mutant alleles of *met30-6* or *cdc4-1*, but are also observed upon depletion of Met30 and Cdc4 or in *MET30* deletion mutants, which are viable in a *MET32* deletion background.

In WT cells, non-centromeric localization of endogenous Cse4 is barely detectable [28, 74, 75], suggesting that there must be mechanisms to ensure that Cse4 is not stably maintained at non-centromeric regions above a certain threshold for chromosomal stability. Here, we define a role for SCF Met30/Cdc4-mediated proteolysis of endogenous Cse4 in preventing its stable maintenance at non-centromeric regions to ensure faithful chromosome segregation. Support for our conclusion is based on several experimental evidences such as higher stability of chromatin associated Cse4, mislocalization of Cse4, plasmid and chromosome loss and defects in kinetochore integrity in *met30-6* and *cdc4-1* strains. We propose that the plasmid/chromosome loss is most likely linked to Cse4 mislocalization in *met30-6* and *cdc4-1* mutants because mislocalization as well as chromosome loss are suppressed by constitutive expression of H3 (Δ*16H3*). Previous studies have shown that Δ*16H3* suppresses mislocalization and chromosome loss in a strain where Cse4 is stabilized by mutating potential ubiquitin acceptor lysines (cse4^16KR^), likely by competing with cse4^16KR^ for incorporation at non-centromeric sites [4]. Similarly, overexpression of histone H3 suppresses chromosome loss defects due to mislocalization of Cnp1 in *S*. *pombe* [69].

The chromosome loss phenotype in *met30-6* and *cdc4-1* strains is not due to reduced levels of Cse4 at centromeres because ChIP-qPCR and ChIP-seq experiments showed that the levels of Cse4 at the *CEN* were actually somewhat higher in *met30-6* and *cdc4-1* than the WT strain. We propose that the increased levels of *CEN* associated Cse4 may be due to higher efficiency of cross-linking of Cse4 to *CEN* chromatin due to defects in kinetochore structure/integrity in *met30-6* or *cdc4-1* strains. Consistent with this hypothesis, we observed defects in the integrity of the native kinetochore in *met30-6* and *cdc4-1* mutants based on reduced protection of the centromeric DNA to digestion by *DraI* endonuclease, similar to that reported for kinetochore mutants [34, 59, 71–73]. Previous studies from fission yeast, fly and human cells have suggested that mislocalization of Cnp1/CID/CENP-A contributes to weakened kinetochores and a CIN phenotype [6, 7, 16, 76, 77]. Future studies will be necessary to understand the mechanistic basis for kinetochore integrity defects caused directly or indirectly by Cse4 mislocalization.

Intriguingly, our results revealed that Met30 and Cdc4 act cooperatively to restrict Cse4 abundance, thereby preventing Cse4 mislocalization. Several experimental evidences support our conclusion, for example a) defects in Cse4 proteolysis in a *met30-6 cdc4-1* strain are similar to that observed in *met30-6* strain, b) interaction between Met30 and Cdc4, and of both proteins with Cse4 *in vivo* and c) defects in the interaction of Cdc4 with Cse4 in *met30* mutants. We propose a model in which the SCF-Met30/Cdc4 heterodimer confers a distinct substrate specificty for ubiquitin-mediated proteolysis of Cse4 thereby preventing its mislocalization to ensure faithful chromosome segregation (Fig 7J). Heterodimerization of two related F-box proteins Pop1 and Pop2 for degradation of cell cycle regulators has been reported in fission yeast [78, 79]. This multimerization is mediated through the N-terminal region, likely through a similar mode as described for D-domain homodimerization [66, 79]. Previous studies have shown that homodimerization of SCF complexes mediated by the D-domain located in the N-terminal region adjacent to the F-box domain is important for their function [65–67]. These homodimers consist of two complete SCF units and are likely required for efficient substrate engagement. Surprisingly, the Met30 D-domain, although essential for viability and ubiquitination of its canonical substrate Met4, is not involved in formation of the SCF-Met30/Cdc4 complex, nor is it required for suppression of *GAL-CSE4* SDL in the *met30-6* strain. We demonstrated that the interaction of Met30 with Cdc4 is mediated by the C-terminal WD40 domain. This is remarkable as it indicates an alternative architecture for SCF complexes with two different F-box proteins. Whether the SCF-Met30/Cdc4 ligase complex contains two components of the SCF core (Cdc53, Skp1, Rbx1) is currently unknown, but dimerization through the WD40 region is unlikely to impede on Cdc53/Skp1 binding to F-box proteins. Our results show that Cse4 substrate recognition depends on Met30 within the SCF-Met30/Cdc4 complex, but Cdc4 clearly plays a critical role in Cse4 proteolysis and in preventing mislocalization of Cse4 for faithful chromosome segregation. Met30 likely drives recognition and binding of Cse4, because we could reconstitute the interaction with recombinant purified SCF-Met30 and Cse4 (S13 Fig), but Cdc4 may recognize an additional protein within the Cse4 complex that could act as a specific marker for the identification and degradation of mislocalized Cse4 from non-centromeric regions. Alternatively, Cdc4 may be required to position SCF-Met30/Cdc4 on Cse4 to stimulate ubiquitin transfer. Future studies will answer these exciting questions uncovered by our results. A role for Met30 that is independent of homodimerization has not been reported so far. From a physiological standpoint the requirement of two proteins may allow fine tuning of cellular levels of Cse4 to prevent its mislocalization and CIN.

In summary, our genome-wide screen provides insights into evolutionarily conserved essential genes that are required for growth when Cse4 is overexpressed. We have shown that the SCF-Met30/Cdc4 pathway is likely the first and perhaps a major pathway responsible for regulating cellular levels of Cse4 thereby defining a critical mechanism by which unperturbed cells ensure high fidelity chromosome segregation. These studies are significant from a clinical standpoint as mislocalization of CENP-A has been observed in numerous cancers and proposed to contribute to aneuploidy and tumorigenesis [9–14, 80]. Human homologs of Met30 (β-TrCP) and Cdc4 (Fbxw7) have also been implicated in cancers. For example, reduced expression of β-TrCP has been reported in lung cancers and high levels of CENP-A are reported in lung adenocarcinoma [81, 82]. Interestingly, Fbxw7 localizes to human chromosome 4q31.3, which is deleted in about 30% of human cancers and somatic mutations in Fbxw7 have been detected in tumors of diverse tissue origin, including blood, breast, bile duct, colon, endometrium, stomach, lung, bone, ovary, pancreas and prostate [83, 84]. Furthermore, loss or depletion of FBWX7 causes CIN and tumorigenesis in human cancers [83, 85]. Based on our results with budding yeast, it is likely that β-TrCP and Fbxw7 may also regulate ubiquitin-mediated proteolysis of CENP-A to prevent its mislocalization and CIN. Our study describes a conserved pathway that ensures chromosomal stability. Future studies will shed light on details of the corresponding human pathways and their roles in tumor development.

## Materials and methods

### Strains, plasmids and growth conditions

The yeast strains and plasmids used in this study are listed in S2 Table. Unless noted otherwise, the yeast strains used are isogenic to BY4741. An SGA query strain (YMB6969) in which the endogenous *CSE4* was replaced by HA-tagged *CSE4* expressed from the *GAL1* promoter was created in Y7092 by homologous recombination [86]. Briefly, two PCR products containing the *GAL1* promoter driving HA-CSE4 and MX4 NatR were obtained using templates pMB1458 and p4339, respectively. The ClonNat resistant transformants that failed to grow in glucose-containing medium, but grew and overexpressed HA-Cse4 on galactose-containing medium were used as SGA query strains. WT, *met30-6* and *cdc4-1* strains expressing *HA-CSE4* under its native promoter at the endogenous locus were created as described above except pRB199 was used as a template for *HA-CSE4*. To generate Met30 degron strains with an auxin-inducible +/- *OS-TIR1* system (YMB9675 and YMB9677) and N-terminal Myc-tagged Cdc4 strain (YMB9674), a *KAN*::*CUP1-Myc-AID* PCR fragment was integrated into the 5’ of *MET30* and *CDC4* genes by homologous recombination. The Myc-Aid-Met30 degradation was induced with auxin as described previously [63, 64]. To generate the *CDC4* shut-off strain (YMB10212), the *KAN*::*pGAL-HA* PCR fragment was integrated into the 5’ of the *CDC4* gene by homologous recombination. To N-terminally HA tag *CDC4* from its endogenous locus, a PCR fragment containing the *CDC4* promoter and HA epitope sequences was transformed into YMB10212 to replace *KAN*::*pGAL* to generate *cdc4Δ*::*HA-CDC4* (YMB10217). Otherwise indicated, all yeast strains used in this study were grown at the permissive temperature of 25°C.

Plasmid pMB1458 expresses *GAL-HIS-HA-CSE4* and pMB1597 expresses *GAL-HA-CSE4* as described previously [19]. To construct pMB1840 with *Flag-CDC4* driven by the *CDC4* promoter, fragments including the *CDC4* promoter, Flag sequence and *CDC4* gene were amplified and assembled into *pCDC4-Flag-CDC4* based on the overlapping sequences of the fragments. *pCDC4-Flag-CDC4* was cloned into pRS425 (*2μ*, *LEU2*) via *SpeI* and *XhoI* restriction sites. pMB1831 carrying *HA-CSE4* driven by the *CSE4* promoter was created by subcloning the *pCSE4-HA-CSE4* fragment from pBR199 into pRS426 (*2μ*, *URA3*) via *HindIII* and *SpeI* sites. Plasmid pMB1830 carrying *pMET30-Myc-met30ΔD-domain* was created by deleting the D-domain sequence in pK699 with Quick Change II Site-Directed Mutagenesis Kit (Agilent). Plasmid pMB1861 with *pMET30-Myc-met30ΔWD40*, fragments upstream and downstream of the WD40 domain sequence were amplified using pK699 as template and assembled into *pMET30-Myc-met30ΔWD40* based on the overlapping sequences of the two fragments. *pMET30-Myc-met30ΔWD40* was cloned into pRS415 (*CEN*, *LEU2*) via *SpeI* and *SacI*. YMB10799 (*met30Δ met32Δ*) was created from YMB8789 (*met30-6*) after sequential deletion of *MET32* and *met30-6*.

### SGA screen

An SGA screen using YMB6969 as a query strain grown on galactose-containing medium was performed at 26°C to examine the synthetic fitness defects in an essential TS array (TSA) caused by Cse4 overexpression. A total of 786 TS alleles and 186 non-essential deletion mutants (for internal calibration of interaction score) were used to mate with the query strain. Mutants linked to the *CSE4* locus and markers in the query strain do not result in genetic interactions and hence, are not included in the S1 Table. The procedures for generating the haploid double mutant array and scoring of genetic interactions were described previously [39, 40, 87, 88].

### Protein stability assays

For strains expressing *GALHACSE4* (pMB1597), cultures were grown to logarithmic phase at 25°C in glucose media, washed, resuspended in raffinose-containing medium for 1–2 hours, followed by addition of 2% galactose for 1–4 hours so that the initial induced levels of Cse4 in WT and mutants were similar. Due to the slow growth and poor induction of *GALCSE4* in the *met30-6* and *cdc4-1* strains we grew these strains for longer time periods in galactose medium when compared to the WT strain as indicated in the figure legends. Protein extracts were prepared using TCA methods as described previously [89] at various time points after addition of 2% glucose and CHX (10–50 μg/ml as indicated) to block protein translation. Equal amounts of protein determined by the Bio-Rad DC protein assay (500–0113, Bio-Rad Inc.) from each sample were resolved on a 4–12% Bis-Tris gel (Invitrogen Inc.) for Western blot analysis. For protein stability of Cse4 expressed from its native promoter, cultures were grown to logarithmic phase at 25°C in glucose media and CHX (50 μg/ml) was added. For cell cycle assays, logarithmically grown cultures at 25°C were treated with alpha factor for G1 arrest (3 μM), hydroxyurea (HU, 0.1 M) for S-phase and nocodazole (20 μg/ml) for G2/M arrest, respectively for 90 to 120 minutes. Cell cycle arrest was confirmed by FACS and microscopic analysis for nuclear morphology as described previously [8, 90]. Anti-HA antibody (12CA5, Roche Inc) was used to detect HA-tagged Cse4, rabbit polyclonal antibodies against histone H3 (ab1791, Abcam) to detect histones. Rabbit polyclonal antibodies against Tub2 were custom-made in our laboratory. Secondary antibodies were HRP-conjugated sheep α-mouse IgG (NA931V, Amersham Biosciences) and HRP-conjugated donkey α-rabbit IgG (NA934V, Amersham Biosciences). Western blots were quantified using the SynGene program (SynGene, Cambridge, UK) or ImageJ [91] software. Protein stability is measured as % remaining (normalized to Tub2) at the indicated time after CHX treatment where the initial amount of protein is set to 100%.

### Co-Immunoprecipitation (Co-IP) experiments

Strains were grown in selective medium with 2% glucose for experiments with genes expressed from their native promoter, whereas strains were grown overnight in selective medium containing 2% raffinose to logarithmic phase, diluted in the same selective medium containing 2% galactose and incubated at 30°C for 4 hours for experiments with genes expressed from the *GAL* promoter. Whole cell extracts were prepared by bead beating using a FastPrep-24 homogenizer (MP Biomedicals) in extraction buffer (40mM Hepes, pH7.5, 350mM NaCl, 0.1% Tween, 10% glycerol, protease inhibitors (P8215, Sigma), 1mM DTT, 1mM PMSF). An equal concentration of protein extracts were incubated with anti-HA agarose (A2095, Sigma) at 4°C overnight. The unbound extract was removed following washes in Tris-buffered saline with Tween-20 (0.1%) (TBST) three times, and the immunoprecipitated proteins were eluted in 2X Laemmli buffer or 10 mM Glutathione 50 mM Tris pH8, respectively. Rabbit anti-Myc (sc789, Santa Cruz Inc), mouse anti-Flag (M2, Sigma) and rabbit anti-HA (H6906, Sigma) antibodies were used in Western blot analysis.

### Ubiquitin affinity pull-down assays

Ub pull-down assays for determining the levels of ubiquitinated Cse4 was performed as described previously [19]. Briefly, cell pellets were collected from logarithmically growing cells, resuspended in lysis buffer (20mM Na_2_HPO_4_, 20mM NaH_2_PO_4_, 50mM NaF, 5mM tetra-sodium pyrophosphate, 10mM beta-glycerolphosphate, 2mM EDTA, 1 mM DTT, 1% NP-40, 5 mM N-Ethylmaleimide, 1mM PMSF and protease inhibitor cocktail (Sigma, cat# P8215)) with an equal volume of glass beads (425–600 μM) and were subjected to beads-beating in a FastPrep-24 homogenizer for generating whole cell lysates. A fraction of the lysate was saved as input and an equal amount of cell lysates from WT and mutant strains were incubated with tandem ubiquitin-binding entities (Agarose-TUBE1, Life Sensors, Inc. Catalog #: UM401) at 4°C overnight. The bound proteins were washed in TBST at room temperature and eluted in 2X Laemmli buffer at 100°C for 10 min. The resulting pulled-down proteins were resolved on 4–12% Bis-Tris gel. Ubiquitinated Cse4 was detected by Western blot analysis using anti-HA antibody (Roche Inc., 12CA5).

### Subcellular fractionation and chromosome spreads

Strains expressing endogenous HA-Cse4 were grown at 25°C to logarithmic phase and subcellular fractionation was performed to assay the stability of Cse4 in whole cell extracts (WCE), soluble and chromatin fractions as described previously [4]. Chromosome spreads were performed as described previously [15, 34]. Immunofluorescence was performed for localization of HA-Cse4 using primary antibody 16B12 Mouse anti-HA (1:2500 dilutions, Covance, Babco; MMS- 101P), followed by a secondary antibody (Cy3 conjugated Goat anti-mouse (1:5000 dilutions, Jackson Immuno-Research Laboratories, Inc., 115165003). To detect co-mislocalization of Mif2 and HA-Cse4, the cells were stained with primary antibodies Rabbit anti-Mif2 (1:1000 dilution, a generous gift from Pam Meluh) and 16B12 Mouse anti-HA, followed by secondary antibodies (Cy2 conjugated Goat anti-rabbit, Cy3 conjugated Goat anti-mouse (Jackson Immuno-Research Laboratories, Inc., 115165003)). Cse4 or Mif2 localize to either one or two nuclear foci and mislocalization was scored only when three or more foci or diffuse staining in the nucleus were observed. As a control we examined the localization of Mtw1-GFP (pMB1059) in live WT, *met30-6* and *cdc4-1* strains. Nuclei were visualized by DAPI staining (1 μg/ml in PBS) and Mif2 and Cse4 were detected by Cy2 (green) and Cy3 (red) fluorescence on an Axioskop 2 (Zeiss) fluorescence microscope equipped with a Plan-APOCHROMAT 63X (Zeiss) oil immersion lens. Image acquisition and processing were performed with the IP Lab version 3.9.9 r3 software (Scanalytics, Inc.). Three biological replicates were performed and at least 200 cells were scored.

### Plasmid retention and chromosome transmission fidelity (*ctf*) assays

For plasmid retention assays, WT, *met30-6* and *cdc4-1* strains containing pRS416 (*CEN/URA3* plasmid) were grown selectively in SC-URA medium. Equal OD_600_ of the selectively grown cells were plated on SC-URA and YPD as T_0_. Equal OD of each strain were inoculated in YPD and allowed to grow for 10 generations (10G) without selection. Equal OD of cells at 10G were plated the same as those for T_0_. Colony number of SC-URA/YPD is calculated as the rate of plasmid retention. For *ctf* assays, *cdc4-1* and *Δ16H3* strains were created by integrating *cdc4-1* (YMB10365) and *HHT1-hhf1Δ/Δ16* (YMB6331) into the YPH1015 strain with reporter chromosome (RC). The *cdc4-1 Δ16H3* strain (YMB10366) was created by integrating the *cdc4-1* mutant allele into YMB6331. Assays for the loss of the RC were done as previously reported [92, 93]. Chromosome loss was calculated by counting the number of half-sectored colonies (at least half red) over the total colonies. At least 1000 colonies of each strain were counted in three biological repeats.

### *DraI* accessibility assay

Yeast nuclei were prepared from WT, *met30-6* and *cdc4-1* strains grown in YPD at the indicated temperature as described previously [71–73]. Equal amount of nuclei were resuspended in *DraI* digestion buffer (1M Sorbitol, 20mM PIPES pH 6.8, 0.1 mM CaCl2, 0.5mM MgCl2 and 1mM PMSF) in the presence or absence of *DraI* (100 U/ml) for 30 min at 37°C. Digestion condition with *DraI* was optimized as described previously [71–73] and stopped by addition of EDTA and SDS to final concentration to 50 mM and 2%, respectively. Genomic DNA was extracted with Phenol/Chloroform and QIAquick PCR purification column (Qiagen Inc.). Equal amount of extracted DNA (100 ng) was used for quantitative real time PCR (qPCR) with primers flanking the *CEN1* and *CEN3* to determine the susceptibility of *CEN* chromatin to *DraI* digestion.

### Chromatin immunoprecipitation (ChIP) sequencing and ChIP-qPCR

Chromatin immunoprecipitation was performed with two biological replicates as described previously [33, 94]. Wild type, *met30-6*, and *cdc4-1* strains expressing HA-Cse4 were grown logarithmically in YPD at 25°C. Cells were cross-linked in formaldehyde (1%) for 15 min at room temperature, and ChIP was performed as described previously [33]. ChIP-qPCR was performed using 7500 Fast Real Time PCR System with Fast SYBR Green Master Mix (Applied Biosystems) using the following conditions: 95°C for 20 sec followed by 40 cycles of 95°C for 3 sec, 60°C for 30 sec. The enrichment was calculated as % input using the _dd_C_T_ method [95]. ChIP-seq libraries for paired-end sequencing were constructed from 50 ng of ChIP and input DNA using a Nextera DNA Library Kit (Illumina Inc.) and details are provided in the legend to Fig 7.

## Supporting information

S1 Fig *met30-6* and *cdc4-1* strains exhibit a normal cell cycle profile at permissive temperature. FACS analysis to measure DNA content of cells was performed with WT (YMB8788), *met30-6* (YMB8789) and *cdc4-1* (YMB9571) strains grown at 25°C in glucose containing media as described in Fig 2.
(TIF)

S2 Fig Cell cycle arrest of WT, *met30-6* and *cdc4-1* strains by α-factor, HU or Nocodazole. Fluorescent Activated Cell Sorting (FACS) analysis was performed with cells arrested with α-factor, HU or Nocodazole for 90 minutes at 25°C used in Fig 4B. Nuclear morphology was used to determine the percentage of cells that show unbudded (G1), small budded (S) and large budded (G2/M) arrest phenotype of cells from A. At least, 100 cells were counted for each strain for each of the arrest.
(TIF)

S3 Fig Endogenous HA-Cse4 is stabilized in Met30 or Cdc4-depleted cells. (A) Endogenous HA-Cse4 is stabilized upon depletion of Met30. Western blot analysis was performed with WCE from a *MET30* degron (AID-tagged *MET30-Myc*) strain expressing HA-Cse4 from the endogenous locus with (YMB9677) or without *OSTIR1-Myc* (YMB9675) grown at 25°C. Depletion of Met30 is triggered by the addition of auxin (1mM) for 2 hours. Western blots were probed with anti-HA or anti-Tub2 antibodies. Percentage of HA-Cse4 remaining at 90 minutes after CHX treatment (50 μg/ml) is shown. (B) Deletion of *MET32* suppresses the temperature sensitivity of *met30Δ* strain. Growth assays with WT (YMB9673), *met30-6* (YMB8789) and two independent *met30Δ met32Δ* (YMB10799) isolates were performed using 5-fold serial dilutions and plated on YPD at either 25°C or 35°C. (C) Cdc4 is depleted in *CDC4* shut-off strain transiently grown in glucose medium. A *CDC4* shut-off strain, *cdc4Δ*::*KAN*::*pGAL-HA-CDC4* (YMB10212), grown in galactose medium was shifted to glucose medium for the indicated times. Depletion of Cdc4 was observed 60 minutes after shift to glucose medium. Western blots were probed with anti-HA or anti-Tub2 (as a loading control) antibodies.
(TIF)

S4 Fig Met30 regulates the interaction of Cdc4 with Cse4 and homodimerization domain of Met30 is dispensable for Cse4 proteolysis. (A) The interaction between Cdc4 and Cse4 is reduced in a *met30Δ met32Δ* strain. Co-IP experiments were performed with anti-HA agarose using WCE from WT strain (YMB9673) expressing *Flag-CDC4* (pMB1840) with or without *HA-CSE4* (pMB1831); *met30Δ met32Δ* strain (YMB10799) expressing *Flag-CDC4* (pMB1840) with or without *HA-CSE4* (pMB1831) grown in selective glucose medium at 25°C. Input and IP (anti-HA) samples were analyzed by Western blot analysis and probed with anti-Flag and anti-HA antibodies. All tagged proteins are expressed from their native promoter. (B) *met30ΔD* fails to suppress the temperature sensitivity of a *met30-6* strain. WT and *met30-6* strains expressing vector (pRS415), *MET30* (pP88) or *met30ΔD* (pMB1918) were grown to logarithmic phase at 25°C and five-fold serial dilutions were plated on glucose plates and incubated at 25°C or 35°C. (C) Homodimerization of Met30 is required for ubiquitination of Met4, and *met30Δ*D does not rescue the ubiquitination defect of Met4 in *met30Δ met32Δ* strain. *met30Δ met32Δ* double mutant strains expressing vector (pRS415), *MET30* (pP88) or *met30ΔD* (pMB1918) were grown to logarithmic phase at 30°C in YPD medium and cell lysates were analyzed by immunoblotting using anti-Met4 antibodies to visualize the Met4 ubiquitination status. Defects in Met4 ubiquitination in *met30Δ met32Δ* strain were not rescued by met30*Δ*D and were similar to that observed with the vector alone.
(TIF)

S5 Fig Defects in ubiquitination of Met4 are observed in *met30*-6 strains but not in *cdc4-1* strain. Western blot analysis of WCE from *met30-6* (PY283) and *cdc4-1* (PY187) grown in YPD to logarithmic phase at 25°C and after a shift to 37°C for 30, 60 or 120 minutes was performed, and blots were probed with anti-Met4 antibodies to visualize the Met4 ubiquitination status.
(TIF)

S6 Fig Mislocalization of Cse4 in *met30-6* and *cdc4*-1 strains. Cse4 expressed from its endogenous locus is mislocalized to non-centromeric chromatin in *met30-6* and *cdc4-1* cells. Representative images from Fig 7A showing that localization of Cse4 restricted to one or two foci in WT cells and mislocalization of Cse4 to a larger area or multiple foci in *met30-6* and *cdc4-1* cells. Blue: DAPI; Magenta: Cse4.
(TIF)

S7 Fig Mutations in *met30-6* and *cdc4-1* contribute increased plasmid loss. (A) Plasmid loss assays were performed using *met30-6* (YMB8789) and *cdc4-1* (YMB9571) strains transformed with WT copy of *MET30* (pMB1619) or *CDC4* (pMB1717), respectively. Plasmid retention is calculated as number of colonies on SC-Ura–Leu/SC-Leu plates after non-selective growth in SC-Leu medium. Three biological repeats were performed with indicated strains. Mean±standard deviation and p value are shown. * p value<0.02 (B) Deletion of *MET32* does not suppress the plasmid loss of *met30Δ* strain. Plasmid loss assays were performed with WT (YMB9673), *met30-6* (YMB8789) and *met30Δ met32Δ* (YMB10799) strains. Plasmid retention is calculated as number of colonies on SC-Ura/YPD plates after non-selective growth in YPD. Three biological repeats with the mean+/- standard deviation are shown. Percentage of plasmid retention is normalized to WT as 100%. *met32Δ met30* strain exhibits significant plasmid retention defect when compared to WT strain (p value = 0.0017).
(TIF)

S8 Fig *Δ16H3* suppresses *GAL-CSE4* SDL and enrichment of Cse4 in chromatin in *met30-6* strain. (A) *Δ16H3* partially suppresses the SDL of *GALCSE4* in *met30-6* strain. Growth assays were performed with WT, *met30-6* (YMB9984), *met30-6 Δ16H3* (YMB9986) strains with GAL-CSE4 (pMB1597) by spotting 5-fold serial dilutions of cells on glucose or galactose plates and incubated at 25°C. (B) *Δ16H3* decreases the stability of endogenous Cse4 in WCE and chromatin in *met30-6* strain. Stability of HA-Cse4 was examined in *met30-6* (YMB11241) and *met30-6 Δ16H3* (YMB11242) strains. % remaining of HA-Cse4 from WCE (4 biological repeats) and chromatin fractions (2 biological repeats) is determined at 45 min post CHX (50 ug/ml) treatment. Tub2 and histone H2B were used to normalize the levels of Cse4 for WCE and chromatin, respectively. Mean+/-standard deviation is shown (WCE). The p value for the effect of *Δ16H3* on Cse4 stability in WCE is <0.05. (C) *Δ16H3* does not suppress the temperature sensitivity of *met30-6* strain. Growth of WT(YMB9983), *met30-6* (YMB9984) *and met30-6 Δ16H3* (YMB9986) were examined by plating five-fold serial dilutions of respective strains on YPD and incubated at the indicated temperatures. Images shown were photographed at day 5 after plating.
(TIF)

S9 Fig Defect in Cse4 proteolysis in *cdc4-1* strain at 33°C. Western blot analysis was performed on WCE from WT (YMB9673) and *cdc4-1*(YMB9571) strains expressing endogenous HA-Cse4 grown to early logarithmic phase of growth at 25°C and after shift to 33°C for four hours. Western blots were probed with anti-HA and anti-Tub2 antibodies. Percentage of remaining HA-Cse4 at 90 minutes after CHX treatment (50 μg/ml) is indicated. Results from two biological experiments are shown as mean ± average deviation.
(TIF)

S10 Fig Enrichment of HA-Cse4 at *CEN* in WT strain expressing HA-Cse4 but not in untagged strain in ChIP-seq experiments. ChIP-seq was performed with WT strain endogenously expressing HA-Cse4. An untagged WT strain was used as a control to determine the levels of background. Representative images of the ChIP-seq results showing enrichment of HA-Cse4 along chromosomes are shown. As expected, HA-Cse4 enrichment was largely detected at the *CEN*s. No significant enrichment was detected in untagged control strain at *CENs* or non-centromeric regions. DNA sequence data are available from the NCBI GEO repository under the accession reference number GSE129195 (https://www.ncbi.nlm.nih.gov/geo/query/acc.cgi?acc=GSE129195). (A) Chromosome 1. (B) Chromosome 3. (C) Chromosome 6. (D) Chromosome 14.
(TIF)

S11 Fig Genomic distribution of HA-Cse4 in WT, *met30-6* and *cdc4-1* strains. Input (IN) and immunoprecipitated (IP) samples from ChIP experiments described in Fig 7G were used for ChIP-seq as follows: Sequencing libraries were prepared using Illumina Nextera DNA Library Kit #FC-121-1031 and 75-base paired ends reads were obtained by multiplexing on a single Illumina NextSeq model 500 run. Reads were aligned to the sacCer3 genome assembly using Bowtie version 1.0.0 with the following parameters: -n2–3 40 -m3—best—strata -S, and peaks were called using MACS version 2.1.1.20160226 in paired-end mode with the following settings: -g 1.21e7—keep-dup auto -B—SPMR. Pileups were generated during peak calling. Correlation between replicates was found to be greater than 0.98 by UCSC wigCorrelate. Peak calls for replicates were merged, and averaged bedGraph files were generated using macs2 cmbreps. Scores are reads per million total reads. (A and B) Representative images showing enrichment of Cse4 along chromosomes 3 and 14 are shown. As expected, Cse4 enrichment was largely detected at the *CEN* locus. (C) Enrichment of Cse4 at the *CEN* regions. Two-way analysis of variance (to account for differences between centromeres) revealed significant differences in the enrichment of *CEN*-associated Cse4 between wild type and mutants (***, P < 0.001; **, P < 0.01). (D) Comparison of Cse4 enrichment in called peaks at *CEN* and non-*CEN* regions. No significant difference between strains was observed in non-*CEN* peak Cse4 enrichment of Cse4. DNA sequence data are available from the NCBI GEO repository under the accession reference number GSE129195 (https://www.ncbi.nlm.nih.gov/geo/query/acc.cgi?acc=GSE129195).
(TIF)

S12 Fig Kinetochore protein Mif2 but not Mtw1-GFP is mislocalized in *met30-6* or *cdc4-1* strains. (A) Mislocalization of Cse4-interacting protein Mif2 in *met30-6* and *cdc4-1* strains. Localization of endogenous Mif2 and HA-Cse4 were examined by chromosome spreads using WT (YMB9673), *cdc4-1* (YMB9571) and *met30-6* (YMB8789) strains grown at 25°C. Localization of Mif2 and Cse4 were determined using Cy2-and Cy3-labeled secondary antibodies, respectively; nuclei by DAPI staining. Localization of Mif2 is restricted to one or two foci in WT cells and mislocalization of Mif2 or Cse4 to a larger area or multiple foci in WT, *met30-6* and *cdc4-1* cells. In addition, mislocalization of Mif2 was examined in cells that show either no mislocalization (Normal) or mislocalization of Cse4 (Cse4 mislocalized). n = number of cells scored. (B) The *CEN* levels of Mif2 are not reduced in *met30-6* and *cdc4-1* strains. Wild type (WT, YMB9673), *met30-6* (YMB8789) and *cdc4-1* (YMB9571) strains were grown in YPD at 25°C to the logarithmic phase and ChIP was performed using α-Mif2 antibodies (a gift from Pam Meluh) as described in materials and methods. Enrichment of Mif2 at *CEN1*, *CEN3* and *ACT1* (negative control) was determined by qPCR and is shown as % input. Results of two biological replicates denoted as 1 and 2 are shown. (C) Kinetochore protein Mtw1 is not mislocalized in *met30-6* and *cdc4-1* strains. Wild type (YMB9673), *met30-6* (YMB8789) and *cdc4-1* (YMB9571) strains with endogenous HA-Cse4 were transformed with Mtw1-GFP (pMB1059) and grown in selective medium at 25°C except *cdc4-1* that was grown at 33°C. Localization of Mtw1-GFP foci was restricted to one to two foci in WT (n = 135), *met30-6* (n = 112) and *cdc4-1* (n = 120) cells.
(TIF)

S13 Fig SCF-Met30 interacts with Cse4 *in vitro*. Components of SCF-Met30 were co-expressed in insect cells and the complex was purified using the Myc-tag on Met30. The yeast histone octamer containing Cse4 was expressed from a polycistronic construct in *E*. *coli* and purified based on 6xHis-tagged H2A followed by gelfiltration. SCF-Met30 was immobilized on anti-myc beads and incubated with the purified octamer. After several wash steps, binding was assessed by immunoblotting.
(TIF)

S1 Table List of TS alleles of essential genes that exhibit genetic interactions with *GAL-CSE4*. The results of the SGA screen with TS alleles of essential genes and deletion of selected non-essential genes when Cse4 is overexpressed. Listed are the TS allele identified, the mutant allele, SGA score, the standard deviation, and *p*-value scores filtered using the intermediate confidence threshold (*p*-value<0.05 and |Score|>0.08). Interactions that met the intermediate threshold of significance are indicated with a “1” while those that did not are indicated with a “0.” For the significant negative interactors, homology is denoted as C: *Caenorhabditis elegans*; D: *Drosophila melanogaster*; M: mouse; and H: Humans. Homolog and the human gene complementing the yeast mutant information is derived from https://yeastmine.yeastgenome.org/yeastmine/.
(XLSX)

S2 Table *S*. *cerevisiae* strains and plasmids used in this study. Strain numbers, genotypes, and the sources they were derived from (references) are provided.
(DOCX)
