# Supplementary figures and images for: Skp, Cullin, F-box (SCF)-Met30 and SCF-Cdc4-Mediated Proteolysis of CENP-A Prevents Mislocalization of CENP-A for Chromosomal Stability in Budding Yeast
Source: PLoS Genet. 2020 Feb 7;16(2):e1008597. doi: 10.1371/journal.pgen.1008597 (PMC7032732; doi:10.1371/journal.pgen.1008597)

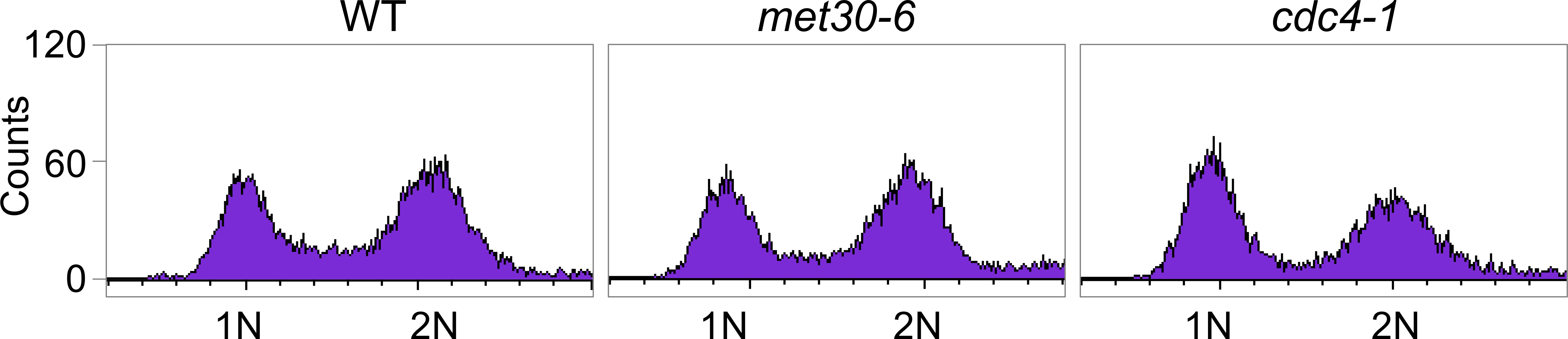

Supplement: S1 Fig — FACS analysis to measure DNA content of cells was performed with WT (YMB8788), met30-6 (YMB8789) and cdc4-1 (YMB9571) strains grown at 25°C in glucose containing media as described in Fig 2. (TIF) [file pgen.1008597.s001.tif]

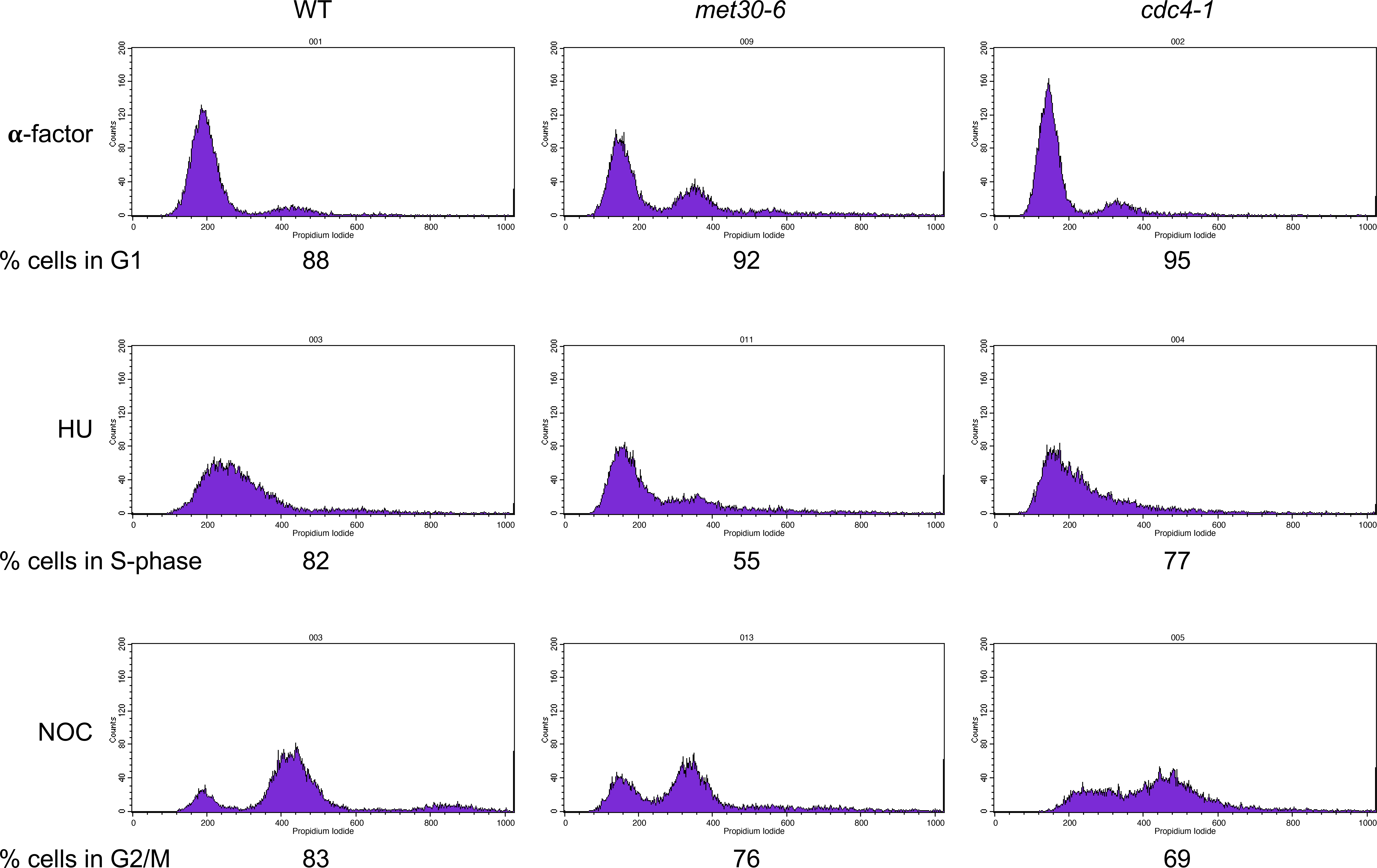

Supplement: S2 Fig — Fluorescent Activated Cell Sorting (FACS) analysis was performed with cells arrested with α-factor, HU or Nocodazole for 90 minutes at 25°C used in Fig 4B. Nuclear morphology was used to determine the percentage of cells that show unbudded (G1), small budded (S) and large budded (G2/M) arrest phenotype of cells from A. At least, 100 cells were counted for each strain for each of the arrest. (TIF) [file pgen.1008597.s002.tif]

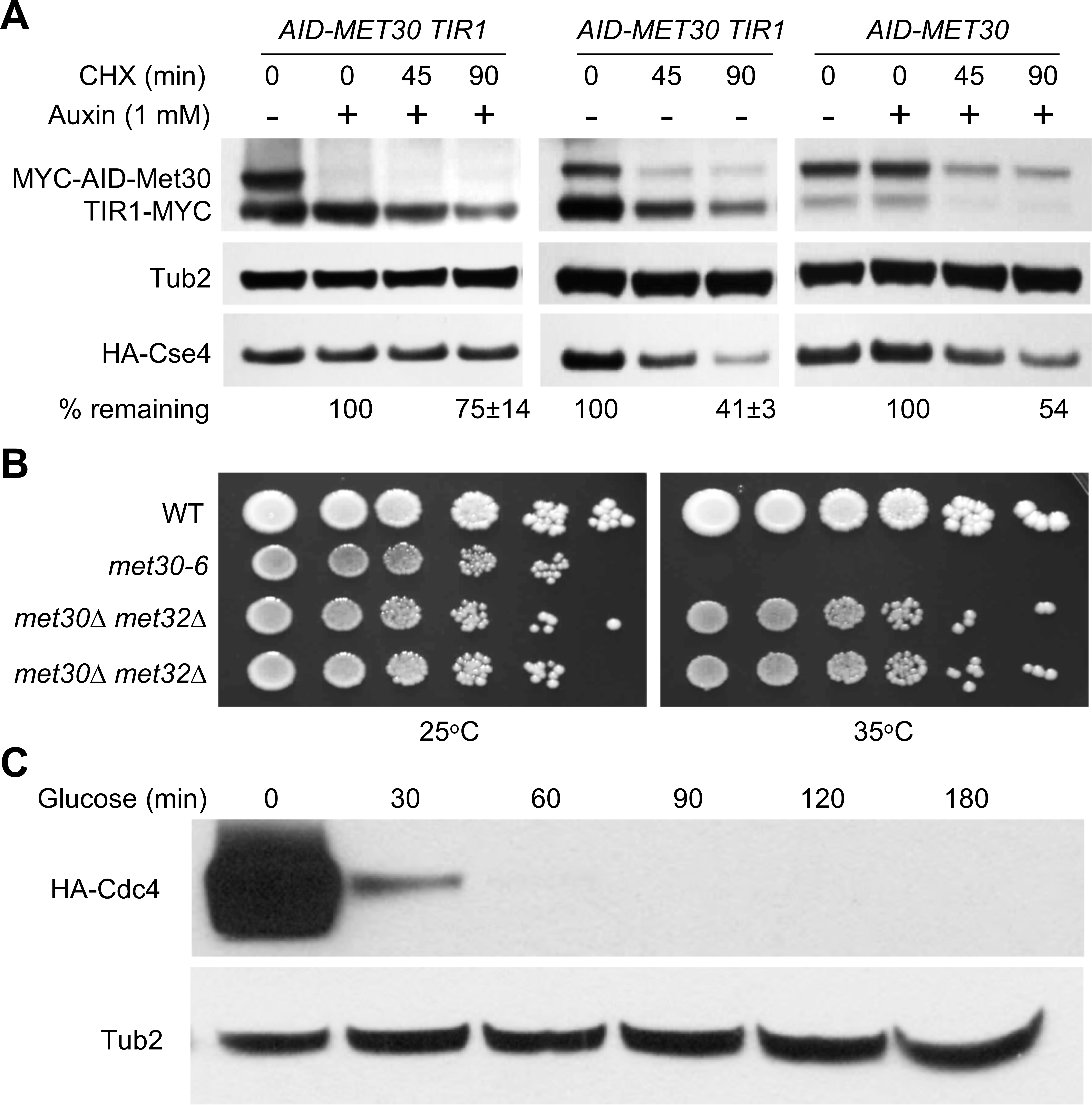

Supplement: S3 Fig — (A) Endogenous HA-Cse4 is stabilized upon depletion of Met30. Western blot analysis was performed with WCE from a MET30 degron (AID-tagged MET30-Myc) strain expressing HA-Cse4 from the endogenous locus with (YMB9677) or without OSTIR1-Myc (YMB9675) grown at 25°C. Depletion of Met30 is triggered by the addition of auxin (1mM) for 2 hours. Western blots were probed with anti-HA or anti-Tub2 antibodies. Percentage of HA-Cse4 remaining at 90 minutes after CHX treatment (50 μg/ml) is shown. (B) Deletion of MET32 suppresses the temperature sensitivity of met30Δ strain. Growth assays with WT (YMB9673), met30-6 (YMB8789) and two independent met30Δ met32Δ (YMB10799) isolates were performed using 5-fold serial dilutions and plated on YPD at either 25°C or 35°C. (C) Cdc4 is depleted in CDC4 shut-off strain transiently grown in glucose medium. A CDC4 shut-off strain, cdc4Δ::KAN::pGAL-HA-CDC4 (YMB10212), grown in galactose medium was shifted to glucose medium for the indicated times. Depletion of Cdc4 was observed 60 minutes after shift to glucose medium. Western blots were probed with anti-HA or anti-Tub2 (as a loading control) antibodies. (TIF) [file pgen.1008597.s003.tif]

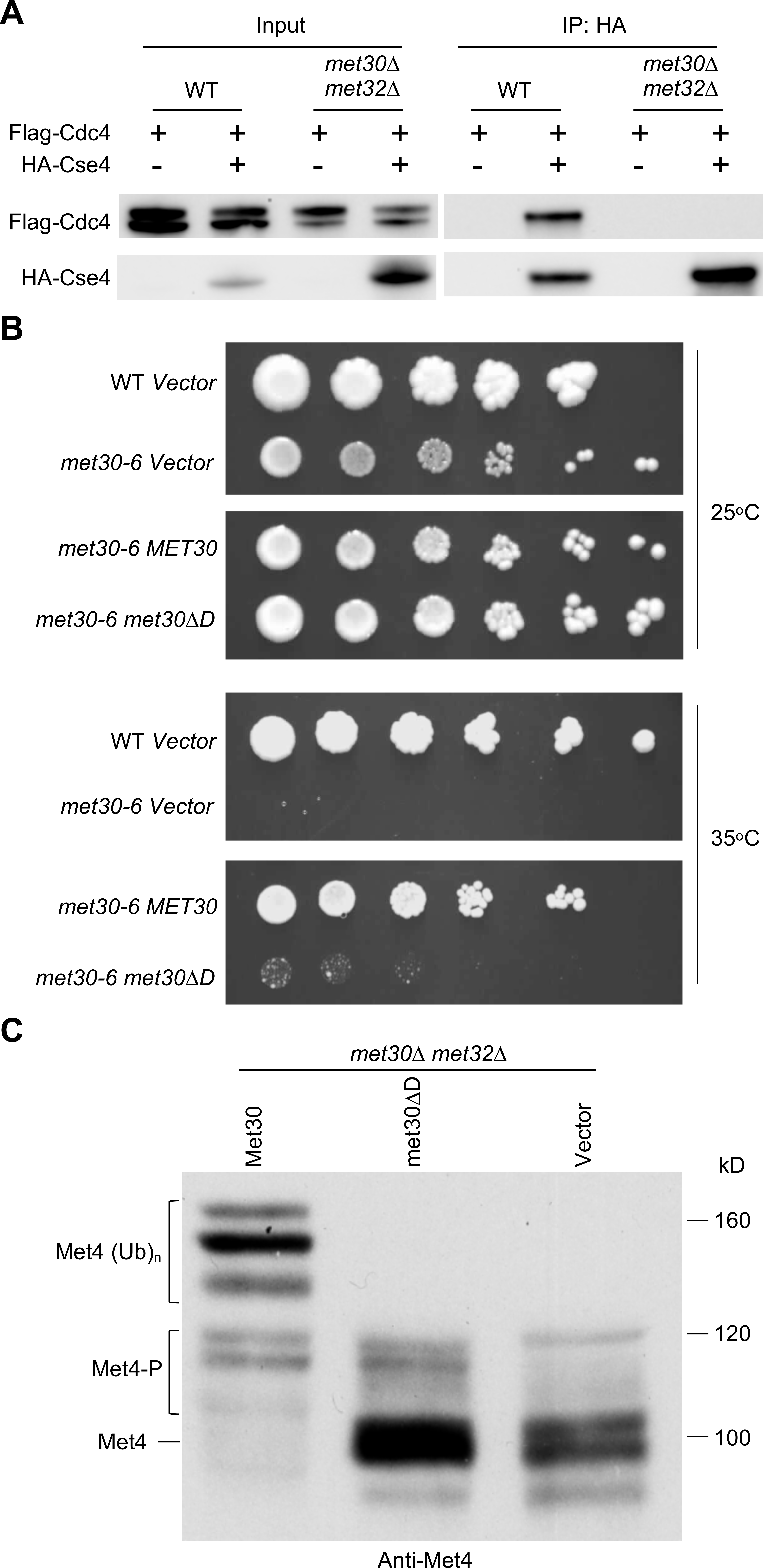

Supplement: S4 Fig — (A) The interaction between Cdc4 and Cse4 is reduced in a met30Δ met32Δ strain. Co-IP experiments were performed with anti-HA agarose using WCE from WT strain (YMB9673) expressing Flag-CDC4 (pMB1840) with or without HA-CSE4 (pMB1831); met30Δ met32Δ strain (YMB10799) expressing Flag-CDC4 (pMB1840) with or without HA-CSE4 (pMB1831) grown in selective glucose medium at 25°C. Input and IP (anti-HA) samples were analyzed by Western blot analysis and probed with anti-Flag and anti-HA antibodies. All tagged proteins are expressed from their native promoter. (B) met30ΔD fails to suppress the temperature sensitivity of a met30-6 strain. WT and met30-6 strains expressing vector (pRS415), MET30 (pP88) or met30ΔD (pMB1918) were grown to logarithmic phase at 25°C and five-fold serial dilutions were plated on glucose plates and incubated at 25°C or 35°C. (C) Homodimerization of Met30 is required for ubiquitination of Met4, and met30ΔD does not rescue the ubiquitination defect of Met4 in met30Δ met32Δ strain. met30Δ met32Δ double mutant strains expressing vector (pRS415), MET30 (pP88) or met30ΔD (pMB1918) were grown to logarithmic phase at 30°C in YPD medium and cell lysates were analyzed by immunoblotting using anti-Met4 antibodies to visualize the Met4 ubiquitination status. Defects in Met4 ubiquitination in met30Δ met32Δ strain were not rescued by met30ΔD and were similar to that observed with the vector alone. (TIF) [file pgen.1008597.s004.tif]

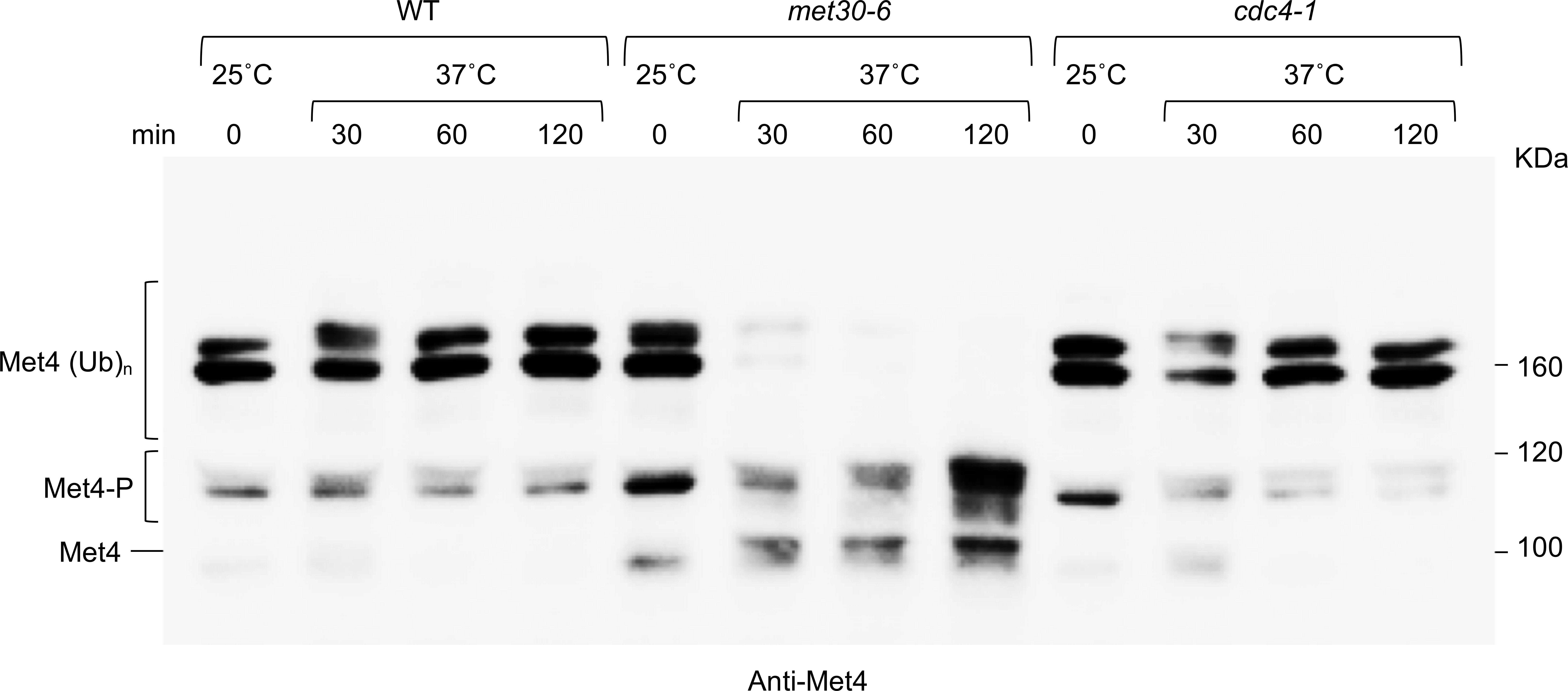

Supplement: S5 Fig — Western blot analysis of WCE from met30-6 (PY283) and cdc4-1 (PY187) grown in YPD to logarithmic phase at 25°C and after a shift to 37°C for 30, 60 or 120 minutes was performed, and blots were probed with anti-Met4 antibodies to visualize the Met4 ubiquitination status. (TIF) [file pgen.1008597.s005.tif]

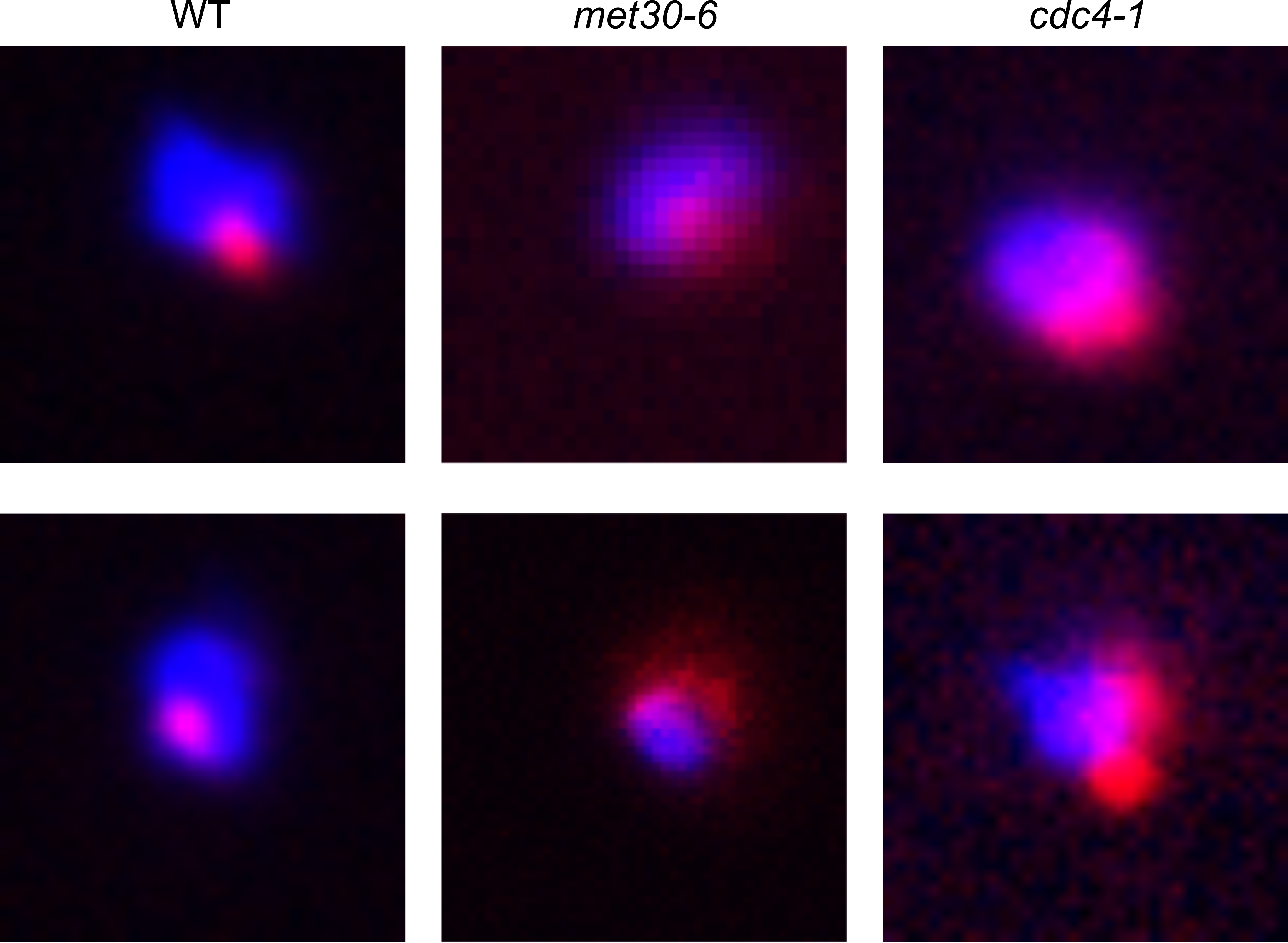

Supplement: S6 Fig — Cse4 expressed from its endogenous locus is mislocalized to non-centromeric chromatin in met30-6 and cdc4-1 cells. Representative images from Fig 7A showing that localization of Cse4 restricted to one or two foci in WT cells and mislocalization of Cse4 to a larger area or multiple foci in met30-6 and cdc4-1 cells. Blue: DAPI; Magenta: Cse4. (TIF) [file pgen.1008597.s006.tif]

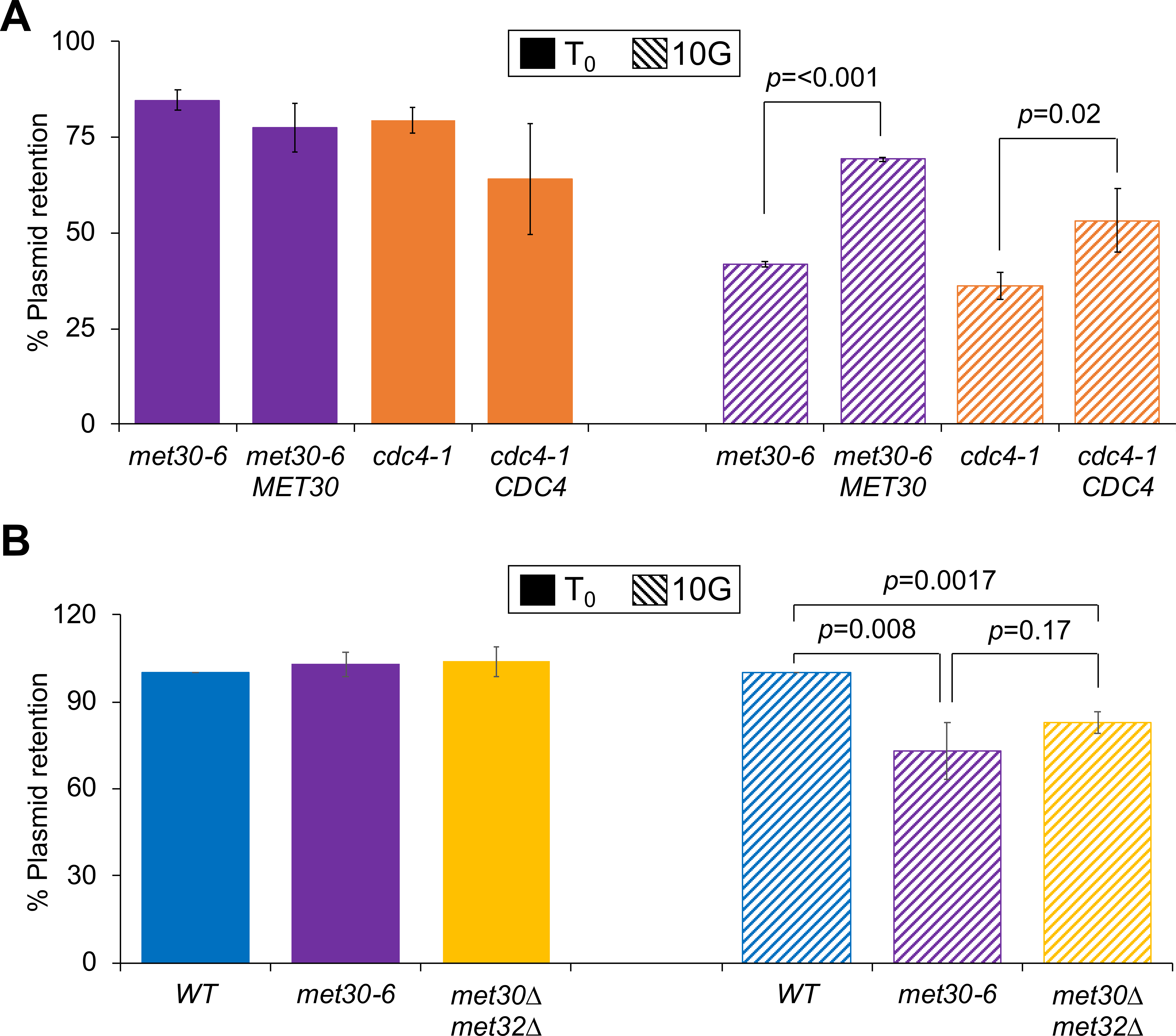

Supplement: S7 Fig — (A) Plasmid loss assays were performed using met30-6 (YMB8789) and cdc4-1 (YMB9571) strains transformed with WT copy of MET30 (pMB1619) or CDC4 (pMB1717), respectively. Plasmid retention is calculated as number of colonies on SC-Ura–Leu/SC-Leu plates after non-selective growth in SC-Leu medium. Three biological repeats were performed with indicated strains. Mean±standard deviation and p value are shown. * p value<0.02 (B) Deletion of MET32 does not suppress the plasmid loss of met30Δ strain. Plasmid loss assays were performed with WT (YMB9673), met30-6 (YMB8789) and met30Δ met32Δ (YMB10799) strains. Plasmid retention is calculated as number of colonies on SC-Ura/YPD plates after non-selective growth in YPD. Three biological repeats with the mean+/- standard deviation are shown. Percentage of plasmid retention is normalized to WT as 100%. met32Δ met30 strain exhibits significant plasmid retention defect when compared to WT strain (p value = 0.0017). (TIF) [file pgen.1008597.s007.tif]

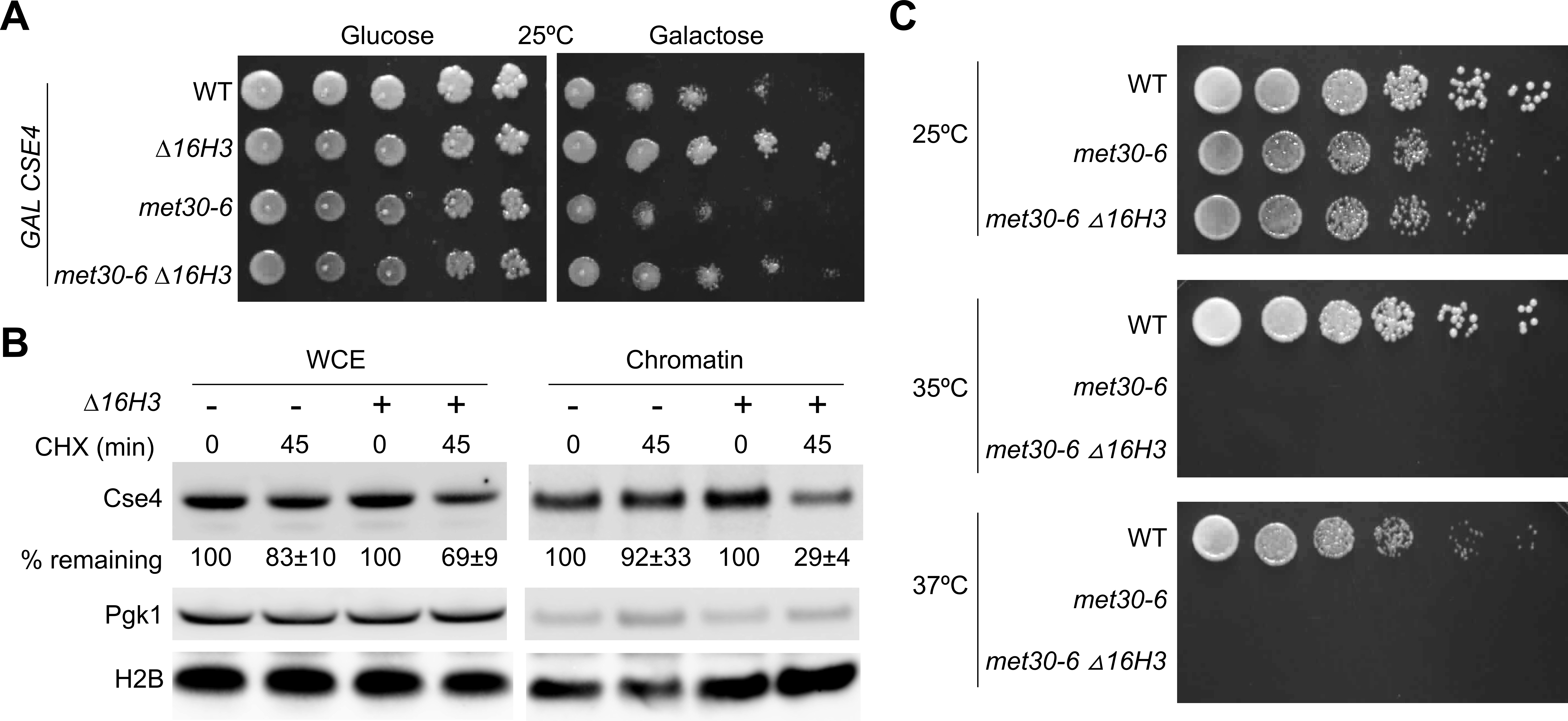

Supplement: S8 Fig — (A) Δ16H3 partially suppresses the SDL of GALCSE4 in met30-6 strain. Growth assays were performed with WT, met30-6 (YMB9984), met30-6 Δ16H3 (YMB9986) strains with GAL-CSE4 (pMB1597) by spotting 5-fold serial dilutions of cells on glucose or galactose plates and incubated at 25°C. (B) Δ16H3 decreases the stability of endogenous Cse4 in WCE and chromatin in met30-6 strain. Stability of HA-Cse4 was examined in met30-6 (YMB11241) and met30-6 Δ16H3 (YMB11242) strains. % remaining of HA-Cse4 from WCE (4 biological repeats) and chromatin fractions (2 biological repeats) is determined at 45 min post CHX (50 ug/ml) treatment. Tub2 and histone H2B were used to normalize the levels of Cse4 for WCE and chromatin, respectively. Mean+/-standard deviation is shown (WCE). The p value for the effect of Δ16H3 on Cse4 stability in WCE is <0.05. (C) Δ16H3 does not suppress the temperature sensitivity of met30-6 strain. Growth of WT(YMB9983), met30-6 (YMB9984) and met30-6 Δ16H3 (YMB9986) were examined by plating five-fold serial dilutions of respective strains on YPD and incubated at the indicated temperatures. Images shown were photographed at day 5 after plating. (TIF) [file pgen.1008597.s008.tif]

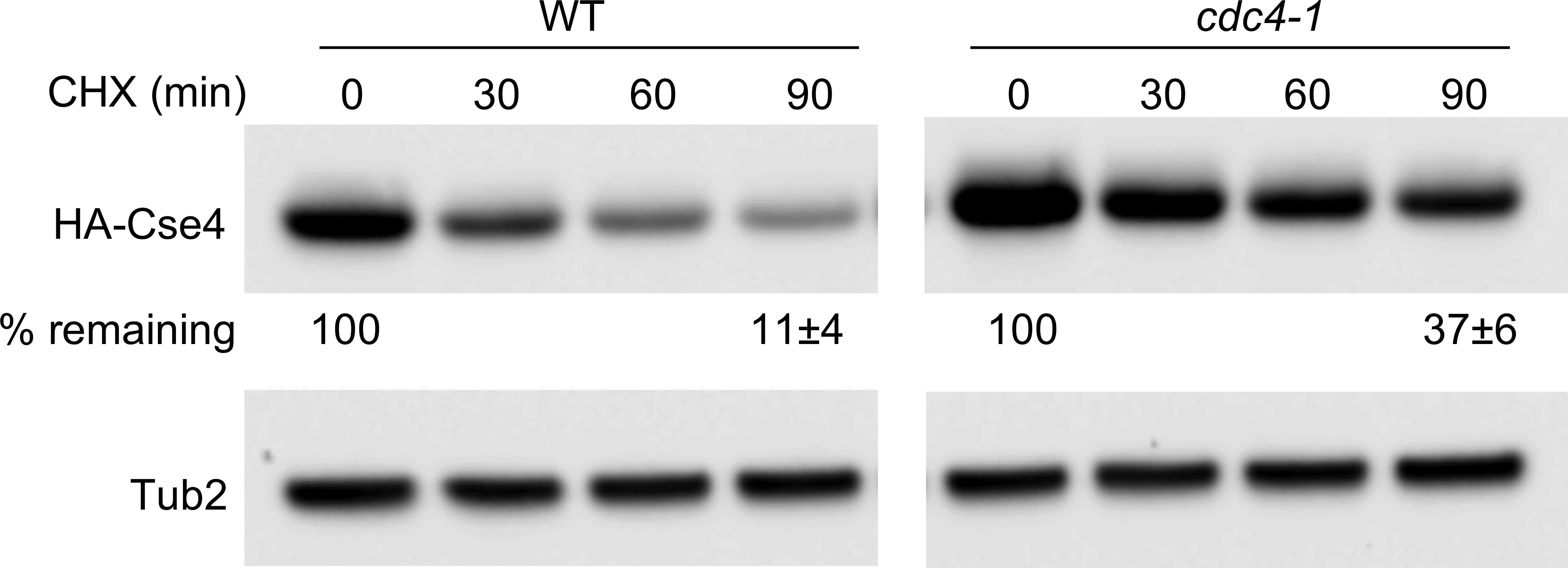

Supplement: S9 Fig — Western blot analysis was performed on WCE from WT (YMB9673) and cdc4-1(YMB9571) strains expressing endogenous HA-Cse4 grown to early logarithmic phase of growth at 25°C and after shift to 33°C for four hours. Western blots were probed with anti-HA and anti-Tub2 antibodies. Percentage of remaining HA-Cse4 at 90 minutes after CHX treatment (50 μg/ml) is indicated. Results from two biological experiments are shown as mean ± average deviation. (TIF) [file pgen.1008597.s009.tif]

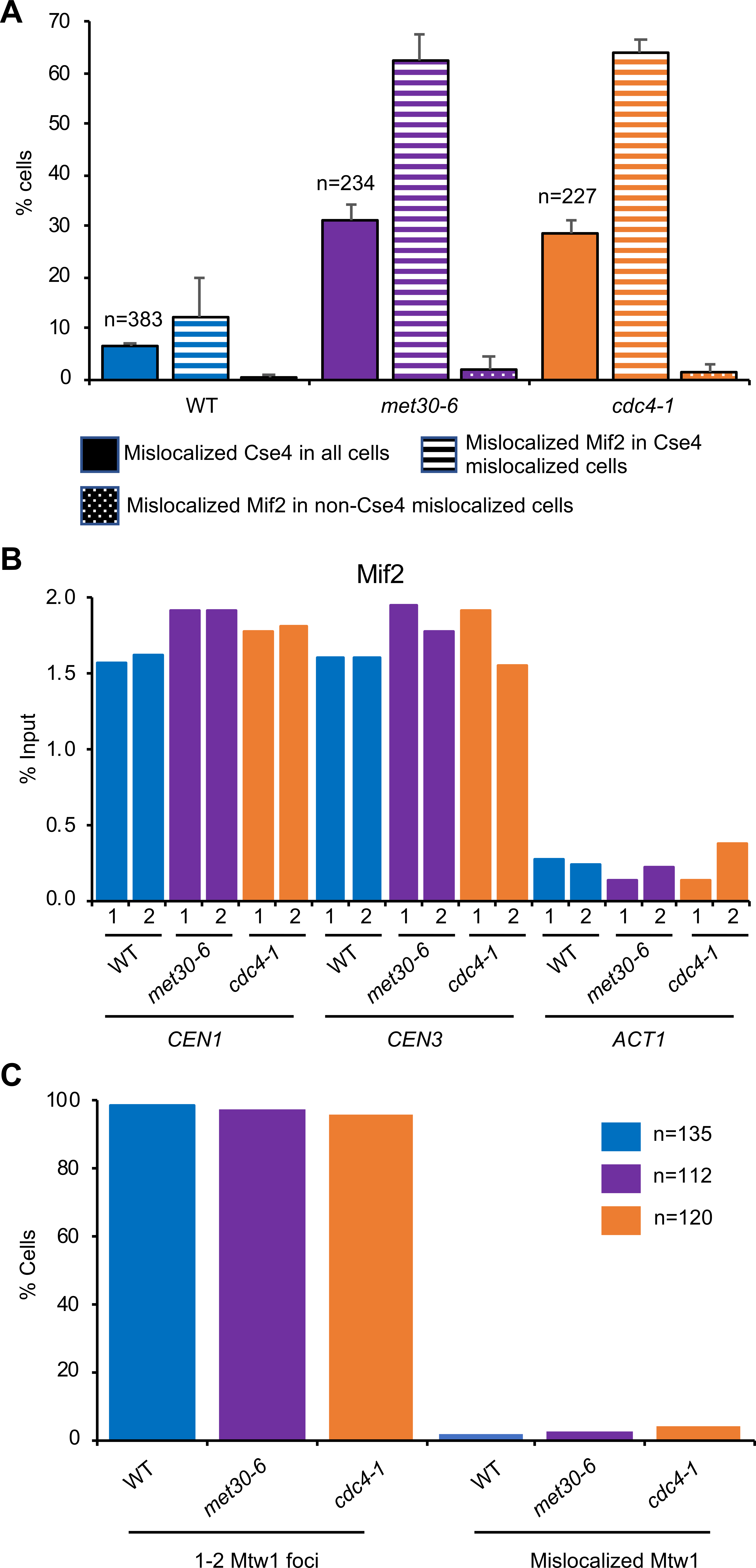

Supplement: S12 Fig — (A) Mislocalization of Cse4-interacting protein Mif2 in met30-6 and cdc4-1 strains. Localization of endogenous Mif2 and HA-Cse4 were examined by chromosome spreads using WT (YMB9673), cdc4-1 (YMB9571) and met30-6 (YMB8789) strains grown at 25°C. Localization of Mif2 and Cse4 were determined using Cy2-and Cy3-labeled secondary antibodies, respectively; nuclei by DAPI staining. Localization of Mif2 is restricted to one or two foci in WT cells and mislocalization of Mif2 or Cse4 to a larger area or multiple foci in WT, met30-6 and cdc4-1 cells. In addition, mislocalization of Mif2 was examined in cells that show either no mislocalization (Normal) or mislocalization of Cse4 (Cse4 mislocalized). n = number of cells scored. (B) The CEN levels of Mif2 are not reduced in met30-6 and cdc4-1 strains. Wild type (WT, YMB9673), met30-6 (YMB8789) and cdc4-1 (YMB9571) strains were grown in YPD at 25°C to the logarithmic phase and ChIP was performed using α-Mif2 antibodies (a gift from Pam Meluh) as described in materials and methods. Enrichment of Mif2 at CEN1, CEN3 and ACT1 (negative control) was determined by qPCR and is shown as % input. Results of two biological replicates denoted as 1 and 2 are shown. (C) Kinetochore protein Mtw1 is not mislocalized in met30-6 and cdc4-1 strains. Wild type (YMB9673), met30-6 (YMB8789) and cdc4-1 (YMB9571) strains with endogenous HA-Cse4 were transformed with Mtw1-GFP (pMB1059) and grown in selective medium at 25°C except cdc4-1 that was grown at 33°C. Localization of Mtw1-GFP foci was restricted to one to two foci in WT (n = 135), met30-6 (n = 112) and cdc4-1 (n = 120) cells. (TIF) [file pgen.1008597.s012.tif]

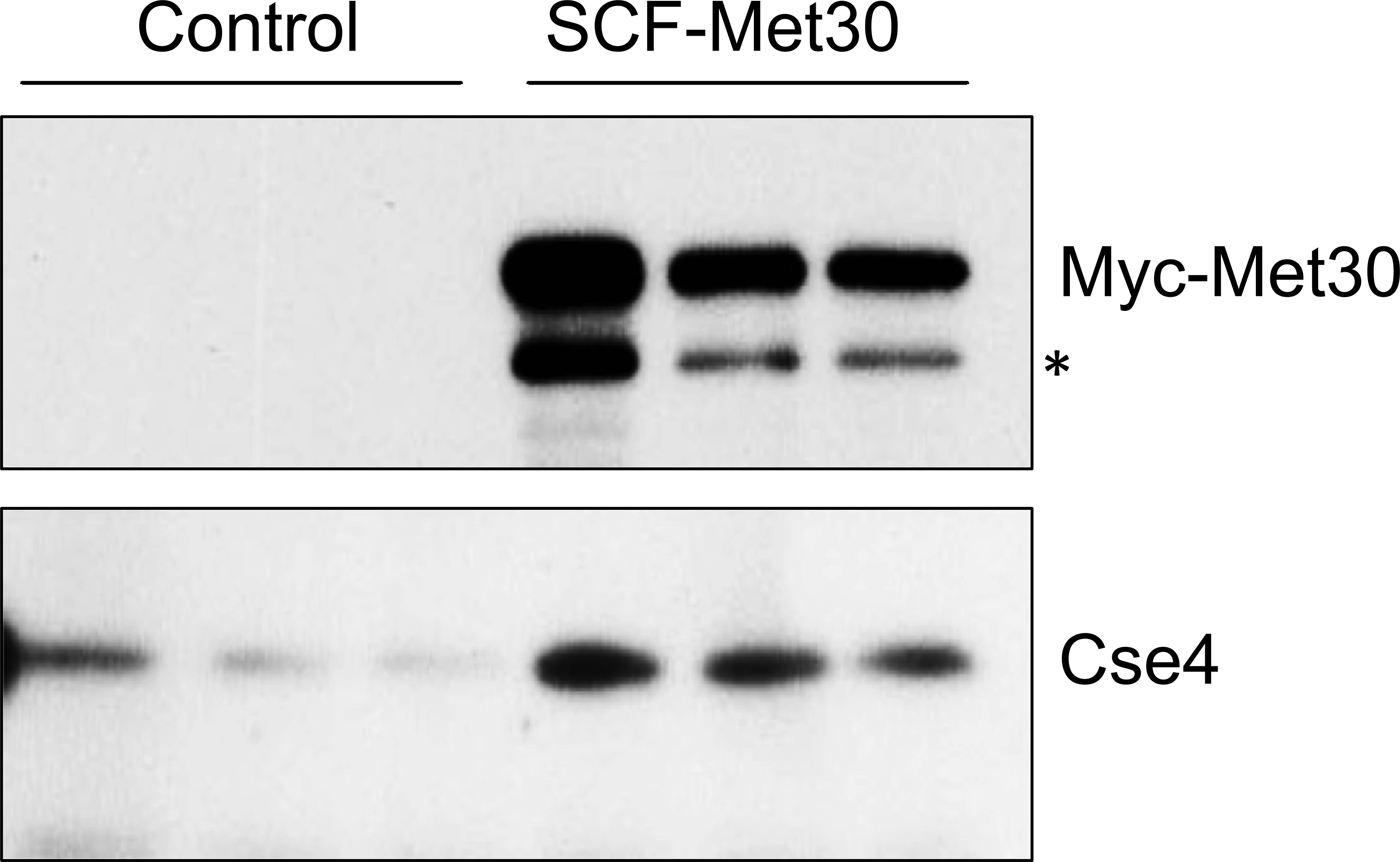

Supplement: S13 Fig — Components of SCF-Met30 were co-expressed in insect cells and the complex was purified using the Myc-tag on Met30. The yeast histone octamer containing Cse4 was expressed from a polycistronic construct in E. coli and purified based on 6xHis-tagged H2A followed by gelfiltration. SCF-Met30 was immobilized on anti-myc beads and incubated with the purified octamer. After several wash steps, binding was assessed by immunoblotting. (TIF) [file pgen.1008597.s013.tif]
