# Supplementary material for: Skp, Cullin, F-box (SCF)-Met30 and SCF-Cdc4-Mediated Proteolysis of CENP-A Prevents Mislocalization of CENP-A for Chromosomal Stability in Budding Yeast
Source: PLoS Genet. 2020 Feb 7;16(2):e1008597. doi: 10.1371/journal.pgen.1008597 (PMC7032732; doi:10.1371/journal.pgen.1008597)
Supplement: S2 Table — Strain numbers, genotypes, and the sources they were derived from (references) are provided. (DOCX) [file pgen.1008597.s015.docx]

**Table S2.** *S. cerevisiae* strains and plasmids used in this study. Strain numbers, genotypes, and the sources they were derived from (references) are provided.

| **List of *S. cerevisiae* strains:** | | |
| --- | --- | --- |
| **Strain** | **Genotype** | **Reference** |
| YMB6331 | *MATa trp1-∆63 lys2-801 leu2-∆1::[HHT1-hhf1∆]∆16::LEU2 ura3-52 ade2-101 his3-∆200 CFIII (CEN3L.YPH278) HIS3 SUP11* | This study |
| YMB6969 | *MATα can1Δ::STE2pr-Sp_his5 lyp1∆ his3∆1 leu2∆0 ura3∆0 met15∆0* LYS2 *cse4∆::pGAL1-CSE4-3HA::NATMX* | This study |
| YMB8788 | *MATα his3Δ1 leu2Δ0 met15Δ0 ura3Δ0 cse4Δ::HA-CSE4::NatR* | This study |
| YMB8789 | *MATa met30-6::KanR his3Δ1 leu2Δ0 met15Δ0 ura3Δ0 cse4Δ::HA-CSE4::NatR* | This study |
| YMB9350 | *MATa his3Δ1 leu2Δ0 met15Δ0 ura3Δ0 met30-6::KAN psh1∆::NatR* | This study |
| YMB9352 | *MATa his3Δ1 leu2Δ0 met15Δ0 ura3Δ0 psh1∆::NatR* | This study |
| YMB9353 | *MATa his3Δ1 leu2Δ0 met15Δ0 ura3Δ0 met30-6::KAN* | This study |
| YMB9337 | *MATα his3 leu2 trp1 ura3 cse4Δ::HA-CSE4::NatR* | This study |
| YMB9571 | *MATa cdc4-1::KAN his3Δ1 leu2Δ0 met15Δ0 ura3Δ0 cse4Δ::HA-CSE4::NatR* | This study |
| YMB9673 | *MATa his3Δ1 leu2Δ0 met15Δ0 ura3Δ0 cse4Δ::HA-CSE4::NatR* | This study |
| YMB9674 | *MATα his3 leu2 trp1 ura3 cse4Δ::HA-CSE4::NatR cdc4∆::KAN::CUP1-Myc-AID-CDC4* | This study |
| YMB9675 | *MATα his3 leu2 trp1 ura3 cse4Δ::HA-CSE4::NatR met30∆::KAN::CUP1-Myc-AID-MET30* | This study |
| YMB9677 | *MATα his3 leu2 OS-TIR1::URA3 trp1 ura3 HA-CSE4::NatR met30∆::KAN::CUP1-Myc-AID-MET30* | This study |
| YMB9713 | *MATa sic1∆::LEU2 cdc4-1::KAN his3Δ1 leu2Δ0 met15Δ0 ura3Δ0 cse4Δ::HA-CSE4::NatR* | This study |
| YMB9983 | *MATa ura3-52 lys2-801 ade2-101 his3∆200 trp1-∆63 leu2-∆1* | This study |
| YMB9984 | *MATa ura3-52 lys2-801 ade2-101 his3∆200 trp1-∆63 leu2-∆1 met30-6::KAN* | This study |
| YMB9985 | *MATa ura3-52 lys2-801 ade2-101 his3∆200 trp1-∆63 leu2∆1::[HHT1-hhf1∆]∆16::LEU2* | This study |
| YMB9986 | *MATa ura3-52 lys2-801 ade2-101 his3∆200 trp1-∆63 leu2-∆1::[HHT1-hhf1∆]∆16::LEU2 met30-6::KAN* | This study |
| YMB10033 | *MATa cdc4-1::KAN met30-6::NatR his3Δ1 leu2Δ0 met15Δ0 ura3Δ0 cse4Δ::HA-CSE4::NatR* | This study |
| YMB10212 | *MATa cdc4∆::KAN::pGAL-HA-CDC4* *his3Δ1 leu2Δ0 met15Δ0 ura3Δ0 cse4Δ::HA-CSE4::NatR* | This study |
| YMB10217 | *MATa cdc4∆::HA-CDC4* *his3Δ1 leu2Δ0 met15Δ0 ura3Δ0* | This study |
| YMB10365 | *MATa cdc4-1::KAN trp1-∆63 lys2-801 leu2-∆1 ura3-52 ade2-101 his3-∆200 CFIII (CEN3L.YPH278) HIS3 SUP11* | This study |
| YMB10366 | *MATa cdc4-1::KAN trp1-∆63 lys2-801 leu2-∆1::[HHT1-hhf1∆]∆16::LEU2 ura3-52 ade2-101 his3-∆200 CFIII (CEN3L.YPH278) HIS3 SUP11* | This study |
| YMB10436 | *MATa trp1-∆63 lys2-801 leu2-∆1 ura3-52 ade2-101 his3-∆200 CFIII (CEN3L.YPH278) HIS3 SUP11 cse4Δ::HA-CSE4::NatR* | This study |
| YMB10437 | *MATa cdc4-1::KAN trp1-∆63 lys2-801 leu2-∆1 ura3-52 ade2-101 his3-∆200 CFIII (CEN3L.YPH278) HIS3 SUP11 cse4Δ::HA-CSE4::NatR* | This study |
| YMB10438 | *MATa trp1-∆63 lys2-801 leu2-∆1::[HHT1-hhf1∆]∆16::LEU2 ura3-52 ade2-101 his3-∆200 CFIII (CEN3L.YPH278) HIS3 SUP11 cse4Δ::HA-CSE4::NatR* | This study |
| YMB10439 | *MATa cdc4-1::KAN trp1-∆63 lys2-801 leu2-∆1::[HHT1-hhf1∆]∆16::LEU2 ura3-52 ade2-101 his3-∆200 CFIII (CEN3L.YPH278) HIS3 SUP11 cse4Δ::HA-CSE4::NatR* | This study |
| YMB10681 | *MATa met32∆::hph met30-6::KAN his3Δ1 leu2Δ0 met15Δ0 ura3Δ0* | This study |
| YMB10859 | *MATa met32Δ::hph his3Δ1 leu2Δ0 met15Δ0 ura3Δ0 cse4Δ::HA-CSE4::NatR* | This study |
| YMB10799 | *MATa met30Δ::HIS3 met32Δ::hph his3Δ1 leu2Δ0 met15Δ0 ura3Δ0 cse4Δ::HA-CSE4::NatR* | This study |
| YMB11241 | *MATa ura3-52 lys2-801 ade2-101 his3∆200 trp1-∆63 leu2-∆1 met30-6::KAN cse4Δ::HA-CSE4::NatR* | This study |
| YMB11242 | *MATa ura3-52 lys2-801 ade2-101 his3∆200 trp1-∆63 leu2-∆1::[HHT1-hhf1∆]∆16::LEU2 met30-6::KAN cse4Δ::HA-CSE4::NatR* | This study |
| YMB11244 | *MATa slx5∆::NatR ubr1∆ psh1∆::LEU2 rcy1∆::KAN ura-* | This study |
| YMB11245 | *MATa slx5∆::NatR ubr1∆ psh1∆::LEU2 rcy1∆::KAN ura- cdc4-1::hph* | This study |
| BY4741 | *MATa his3Δ1 leu2Δ0 met15Δ0 ura3Δ0* | Open Biosystems |
| PK1702 | *MATa bar1∆ ura3∆ns ade1 his2 leu2-3112 trp1-1 Rbx1-13Myc::KAN* | Peter Kaiser |
| TSA878 | *MATa cdc4-1::KAN his3Δ1 leu2Δ0 met15Δ0 ura3Δ0* | [1] |
| *TSA948* | *MATa met30-6::KAN his3Δ1 leu2Δ0 met15Δ0 ura3Δ0* | [1] |
| TSA92 | *MATa cdc34-3::KAN his3Δ1 leu2Δ0 met15Δ0 ura3Δ0* | [1] |
| YPH1015 | *MATa trp1-∆63 lys2-801 leu2-∆1 ura3-52 ade2-101 his3-∆200 CFIII (CEN3L.YPH278) HIS3 SUP11* | [2] |
| Y7092 | *MATα can1Δ::STE2pr-Sp_his5 lyp1∆ his3∆1 leu2∆0 ura3∆0 met15∆0* LYS2 | [1] |
| PY187 | *MATa cdc4-3 bar1* | [3] |
| PY283 | *MATa met30-6::KAN bar1* | [4] |
| YHR333 | *MATa slx5∆::NatR ubr1∆::URA3 psh1∆::LEU2 rcy1∆::KAN* | [5] |

| **List of Plasmids:** | | |
| --- | --- | --- |
| Plasmid Name | Promoter, Gene, marker | Source (Reference) |
| pMB430 | *pGAL Vector* *(2μ, LEU2)* | [6] |
| pMB433 | *pGAL Vector* *(2μ, URA3)* | [6] |
| pMB1059 | *MTW1-GFP (LEU2)* | [7] |
| pMB1458 | *pGAL-His(6X)-3HA-CSE4 (2μ, URA3)* | [8] |
| pMB1597 | *pGAL-3HA-CSE4 (2μ, URA3)* | [8] |
| pMB1617 | *CDC4 MoBY (2μ, LEU2)* | [9] |
| pMB1618 | *CDC34, MoBY (2μ, LEU2)* | [9] |
| pMB1619 | *MET30, MoBY (2μ, LEU2)* | [9] |
| pMB1807 | *pGAL-3HA-CSE4 (2μ, LEU2)* | This study |
| pMB1830 | *pMET30-12Myc-met30 ∆D (CEN, LEU2)* | This study |
| pMB1831 | *pCSE4-3HA-CSE4 (2μ, URA3)* | This study |
| pMB1840 | *pCDC4-Flag-CDC4 (2μ, LEU2)* | This study |
| pMB1861 | *pMET30-12Myc-met30 ∆WD40 (CEN, LEU2)* | This study |
| pMB1892 | *pGAL-3HA-cse4^(16KR)^ (2μ, URA3)* | This study |
| pMB1918 | *pmet30∆D (CEN, URA3)* | This study |
| pMB1951 | *pmet30∆D (CEN, HIS3)* | This study |
| p4339 | *MX4 NatR* | Charles Boone |
| pP88 | *pMET30 (CEN, URA3)* | This study |
| pP1200 | *pMET30-12Myc-met30 ∆77 (CEN, LEU2)* | Peter Kaiser |
| pP1201 | *pMET30-12Myc-met30 ∆113 (CEN, LEU2)* | Peter Kaiser |
| pP680 | *pMET30-12Myc-met30 ∆F-box (CEN, LEU2)* | Peter Kaiser |
| pP699 | *pMET30-12Myc-MET30 (CEN, LEU2)* | Peter Kaiser |
| pRB199 | *pCSE4-6HIS-3HA-CSE4 (CEN, URA3)* | Richard Baker |
| pSB816 | *pGAL-13Myc-CSE4 (2μ, URA3)* | [10] |

1. Tong AH, Boone C. Synthetic genetic array analysis in Saccharomyces cerevisiae. Methods Mol Biol. 2006;313:171-92. Epub 2005/08/25. doi: 1-59259-958-3:171 [pii]. PubMed PMID: 16118434.

2. Spencer F, Gerring SL, Connelly C, Hieter P. Mitotic chromosome transmission fidelity mutants in Saccharomyces cerevisiae. Genetics. 1990;124(2):237-49. Epub 1990/02/01. PubMed PMID: 2407610; PubMed Central PMCID: PMCPMC1203917.

3. Yen JL, Su NY, Kaiser P. The yeast ubiquitin ligase SCFMet30 regulates heavy metal response. Mol Biol Cell. 2005;16(4):1872-82. Epub 2005/02/04. doi: 10.1091/mbc.e04-12-1130. PubMed PMID: 15689486; PubMed Central PMCID: PMCPMC1073668.

4. Kaiser P, Sia RA, Bardes EG, Lew DJ, Reed SI. Cdc34 and the F-box protein Met30 are required for degradation of the Cdk-inhibitory kinase Swe1. Genes Dev. 1998;12(16):2587-97. Epub 1998/08/26. doi: 10.1101/gad.12.16.2587. PubMed PMID: 9716410; PubMed Central PMCID: PMCPMC317080.

5. Cheng H, Bao X, Gan X, Luo S, Rao H. Multiple E3s promote the degradation of histone H3 variant Cse4. Sci Rep. 2017;7(1):8565. Epub 2017/08/19. doi: 10.1038/s41598-017-08923-w. PubMed PMID: 28819127; PubMed Central PMCID: PMCPMC5561092.

6. Mumberg D, Muller R, Funk M. Regulatable promoters of Saccharomyces cerevisiae: comparison of transcriptional activity and their use for heterologous expression. Nucleic Acids Res. 1994;22(25):5767-8. Epub 1994/12/25. PubMed PMID: 7838736; PubMed Central PMCID: PMC310147.

7. Kerscher O, Crotti LB, Basrai MA. Recognizing chromosomes in trouble: association of the spindle checkpoint protein Bub3p with altered kinetochores and a unique defective centromere. Mol Cell Biol. 2003;23(18):6406-18. Epub 2003/08/29. PubMed PMID: 12944469; PubMed Central PMCID: PMCPMC193694.

8. Au WC, Dawson AR, Rawson DW, Taylor SB, Baker RE, Basrai MA. A novel role of the N terminus of budding yeast histone H3 variant Cse4 in ubiquitin-mediated proteolysis. Genetics. 2013;194(2):513-8. Epub 2013/03/26. doi: 10.1534/genetics.113.149898. PubMed PMID: 23525333; PubMed Central PMCID: PMCPMC3664860.

9. Ho CH, Magtanong L, Barker SL, Gresham D, Nishimura S, Natarajan P, et al. A molecular barcoded yeast ORF library enables mode-of-action analysis of bioactive compounds. Nat Biotechnol. 2009;27(4):369-77. Epub 2009/04/08. doi: 10.1038/nbt.1534. PubMed PMID: 19349972; PubMed Central PMCID: PMCPMC3856559.

10. Collins KA, Furuyama S, Biggins S. Proteolysis contributes to the exclusive centromere localization of the yeast Cse4/CENP-A histone H3 variant. Curr Biol. 2004;14(21):1968-72. Epub 2004/11/09. doi: 10.1016/j.cub.2004.10.024. PubMed PMID: 15530401.
